# A novel Na_v_1.5-dependent feedback mechanism driving glycolytic acidification in breast cancer metastasis

**DOI:** 10.1038/s41388-024-03098-x

**Published:** 2024-07-25

**Authors:** Theresa K. Leslie, Aurelien Tripp, Andrew D. James, Scott P. Fraser, Michaela Nelson, Nattanan Sajjaboontawee, Alina L. Capatina, Michael Toss, Wakkas Fadhil, Samantha C. Salvage, Mar Arias Garcia, Melina Beykou, Emad Rakha, Valerie Speirs, Chris Bakal, George Poulogiannis, Mustafa B. A. Djamgoz, Antony P. Jackson, Hugh R. Matthews, Christopher L-H Huang, Andrew N. Holding, Sangeeta Chawla, William J. Brackenbury

**Affiliations:** 1 Department of Biology, University of York, York, UK; 2 York Biomedical Research Institute, University of York, York, UK; 3 Division of Cancer Biology, Institute of Cancer Research, London, UK; 4 Department of Life Sciences, Imperial College London, London, UK; 5 Department of Pathology, School of Medicine, University of Nottingham, Nottingham, UK; 6 Department of Biochemistry, University of Cambridge, Cambridge, UK; 7 Department of Electrical and Electronic Engineering, Imperial College London, London, UK; 8 Institute of Medical Sciences, University of Aberdeen, Aberdeen, UK; 9 Biotechnology Research Centre, Cyprus International University, Haspolat, TRNC, Mersin, Turkey; 10 Physiological Laboratory, University of Cambridge, Cambridge, UK

**Keywords:** Breast cancer, Cancer microenvironment

## Abstract

Solid tumours have abnormally high intracellular [Na^+^]. The activity of various Na^+^ channels may underlie this Na^+^ accumulation. Voltage-gated Na^+^ channels (VGSCs) have been shown to be functionally active in cancer cell lines, where they promote invasion. However, the mechanisms involved, and clinical relevance, are incompletely understood. Here, we show that protein expression of the Na_v_1.5 VGSC subtype strongly correlates with increased metastasis and shortened cancer-specific survival in breast cancer patients. In addition, VGSCs are functionally active in patient-derived breast tumour cells, cell lines, and cancer-associated fibroblasts. Knockdown of Na_v_1.5 in a mouse model of breast cancer suppresses expression of invasion-regulating genes. Na_v_1.5 activity increases ATP demand and glycolysis in breast cancer cells, likely by upregulating activity of the Na^+^/K^+^ ATPase, thus promoting H^+^ production and extracellular acidification. The pH of murine xenograft tumours is lower at the periphery than in the core, in regions of higher proliferation and lower apoptosis. In turn, acidic extracellular pH elevates persistent Na^+^ influx through Na_v_1.5 into breast cancer cells. Together, these findings show positive feedback between extracellular acidification and the movement of Na^+^ into cancer cells which can facilitate invasion. These results highlight the clinical significance of Na_v_1.5 activity as a potentiator of breast cancer metastasis and provide further evidence supporting the use of VGSC inhibitors in cancer treatment.

## Introduction

Breast cancer is the leading cause of cancer-related deaths in women worldwide [1] and most deaths are due to metastatic disease resulting from poor treatment options and therapy resistance [2]. Around 20–30% of patients with primary breast cancer will go on to develop distant metastasis and once this has been diagnosed, there is currently no cure available. Thus, there is an urgent need for improved treatments to prevent or reduce breast cancer metastasis.

Increasing evidence points to ion channels as key regulators of cancer progression [3–6]. Members of the voltage-gated Na^+^ channel (VGSC) family are upregulated in multiple cancer types [7]. In solid cancers, including breast, prostate, lung, and colon cancer, VGSC activity promotes cellular invasion [8, 9]. In breast cancer, the Na_v_1.5 subtype is upregulated at the mRNA level compared to normal tissue and is associated with recurrence and metastasis [10]. Na_v_1.5 is also upregulated in breast cancers at the protein level [11, 12], predominantly in its neonatal D1:S3 splice form [13]; however, the sample sizes of these studies were too small to reliably determine the relationship between Na_v_1.5 expression and clinical outcome. Electrophysiological methods have not yet been used to investigate functional Na_v_1.5 activity in breast cancer tissue or primary cell cultures. Nonetheless, Na^+^ currents carried by Na_v_1.5 have been detected in a small number of breast cancer cell lines and in tissue slices from murine tumour xenografts [11, 12, 14, 15]. In these cells, the persistent Na^+^ current (as distinct from the transient, inactivating Na^+^ current), which passes through the channels at the resting membrane potential (*V*_m_), has been shown to potentiate cellular invasion in vitro and tumour growth and metastasis in vivo [10–12, 14, 16, 17]. Importantly, the metastasis-promoting function of Na_v_1.5 can be inhibited in preclinical models using VGSC blockers, including phenytoin and ranolazine, suggesting that Na_v_1.5 may represent a novel anti-metastatic target for therapeutic intervention [17, 18]. Furthermore, peri-operative administration of the VGSC blocker lidocaine has recently been shown to significantly improve disease-free survival in women with early breast cancer [19].

The mechanism by which VGSCs increase the invasion of cancer cells is incompletely understood [8]. The inward Na^+^ gradient created by the Na^+^/K^+^ ATPase (NKA), a major consumer of cellular ATP [20, 21], is used to power many important functions such as nutrient import and pH regulation [22]. Thus, it would seem wasteful for cancer cells to deplete this inward gradient via Na_v_1.5 upregulation. However, it has been shown that Na^+^ influx via Na_v_1.5 leads to extracellular acidification via the Na^+^/H^+^ exchanger, NHE1, activating pH-dependent cathepsins and promoting invasion [17, 23, 24]. Because an increase in cytosolic [Na^+^] reduces the Na^+^ electrochemical gradient powering H^+^ extrusion via NHE1, the effect of Na_v_1.5 on NHE1 cannot be explained by physical means so an allosteric interaction between the two transporters has been proposed to explain the Na_v_1.5-dependent increase in H^+^ extrusion by NHE1 [23]. An alternative possibility is that Na^+^ influx through Na_v_1.5, rather than the Na_v_1.5 protein itself, is responsible indirectly for increasing H^+^ extrusion through NHE1 and other pH regulators.

In this study, we aimed to delineate the relationship between Na_v_1.5 protein expression and clinical outcome in a large cohort of breast cancer patients. We record Na^+^ currents from patient tissue samples and primary cell cultures for the first time. We also sought to understand the mechanism by which Na_v_1.5 promotes invasion through studying the relationship between channel activity and extracellular acidification.

## Results

### Na_v_1.5 protein expression associates with poor clinical outcomes in breast cancer patients

**Fig. 1:**
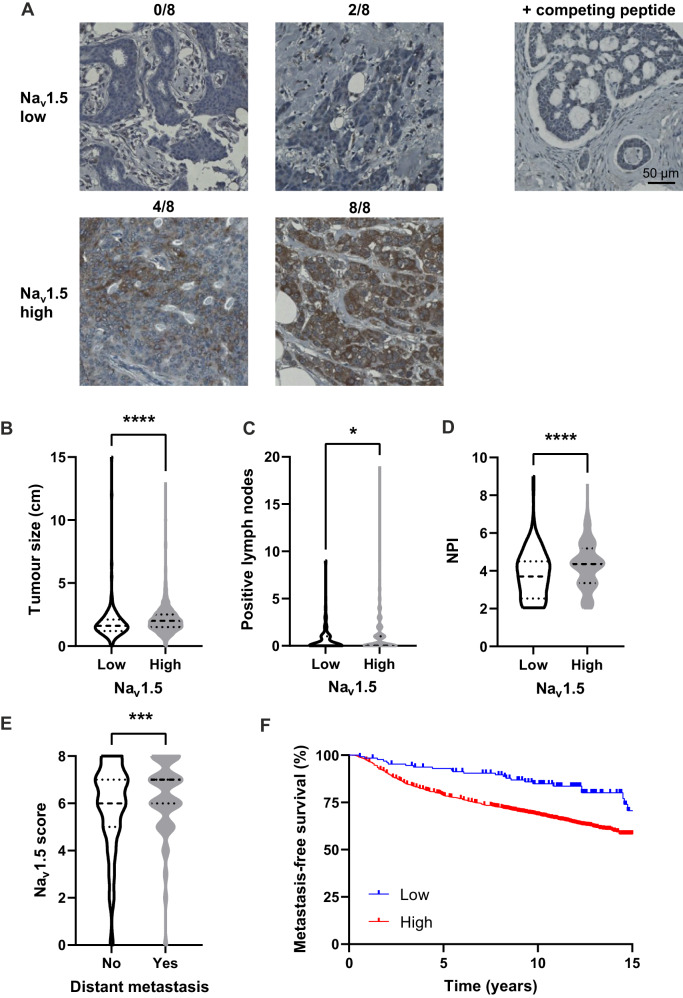
Na_v_1.5 expression in a breast cancer tissue microarray. **A** Examples of low and high Na_v_1.5 staining in carcinoma cells scored using a modified Allred system. Right: staining using anti-Na_v_1.5 antibody which had been preincubated with the immunising peptide. **B** Tumour size compared to Na_v_1.5 score. **C** Number of affected lymph nodes compared to Na_v_1.5 score. **D** Nottingham prognostic index (NPI) compared to Na_v_1.5 score. **E** Na_v_1.5 score in patients with or without recorded distant metastasis. Data are median + quartiles. *P < 0.05, ***P < 0.001, ****P < 0.0001; Mann–Whitney *U* tests (*n* = 1740). **F** Metastasis-free survival compared to Na_v_1.5 score. HR = 2.18 (95% CI 1.63–2.92); *P* < 0.001; log-rank test.

Expression of Na_v_1.5 protein in breast cancer has previously been demonstrated in a small, qualitative study of 6 patients [12] and later in a study of 36 patients [11]. To test the prognostic value of Na_v_1.5 in a larger cohort of patients, we used a breast cancer tissue microarray (TMA) containing 1740 cases. Specificity of staining was confirmed by pre-incubation with the immunising peptide (Fig. 1A). To explore correlation between Na_v_1.5 expression and histoclinical characteristics of the patient population, staining scores between 0 and 3 were classed as ‘low’ and scores between 4 and 8 were classed as ‘high’ (Fig. 1A).

High Na_v_1.5 protein expression was correlated with larger tumour size (*P* < 0.001; Mann–Whitney *U* test; Fig. 1B), lymph node positivity (*P* < 0.05; Mann–Whitney *U* test; Fig. 1C), higher Nottingham prognostic index (*P* < 0.001; Mann–Whitney *U* test; Fig. 1D), and higher tumour grade (*P* < 0.001; *χ*^2^ test; Table 1). In addition, Na_v_1.5 expression was significantly higher in patients who developed a distant metastasis (*P* < 0.001; Mann–Whitney *U* test; Fig. 1E). Na_v_1.5 was negatively associated with estrogen receptor (ER; *P* < 0.05; Fisher’s exact test; Table 1) and progesterone receptor (PgR; *P* < 0.001; Fisher’s exact test; Table 1) expression, but positively associated with human epidermal growth factor receptor 2 (HER2; *P* < 0.01; Fisher’s exact test; Table 1). There was no association between Na_v_1.5 expression and triple-negative breast cancer (TNBC; *P* = 0.12; Fisher’s exact test; Table 1), age (*P* = 0.52; Fisher’s exact test; Table 1), menopause (*P* = 0.46; Fisher’s exact test; Table 1) or endocrine therapy (*P* = 0.51; Fisher’s exact test; Table 1). However, high Na_v_1.5 expression was correlated with recorded chemotherapy use (*P* < 0.05; Fisher’s exact test; Table 1). These relationships are also displayed as violin plots in Supplementary Fig. 1A–L.

**Table 1:** Patient histoclinical characteristics and Na_v_1.5 expression in the tissue microarray.

Variable	Na_v_1.5 expression		*P*
	Low (%)	High (%)	
All cases			
ER			
−	24 (1.6)	348 (23.5)	<0.05
+	113 (7.6)	997 (67.3)	
PR			
−	34 (2.4)	539 (38.2)	<0.001
+	94 (6.7)	744 (52.7)	
HER2			
−	123 (8.8)	1106 (78.7)	<0.01
+	7 (0.5)	170 (12.1)	
TNBC			
−	113 (7.9)	1065 (74.4)	0.12
+	16 (1.1)	238 (16.6)	
Age			
≤50	58 (3.9)	530 (35.9)	0.52
>50	78 (5.3)	811 (54.9)	
Menopause status			
Pre-menopause	58 (4.0)	515 (35.2)	0.46
Post-menopause	79 (5.4)	813 (55.5)	
Lymph node status			
−	95 (6.5)	807 (54.9)	<0.05
+	42 (2.9)	527 (35.8)	
Grade			
1	42 (2.9)	209 (14.3)	<0.001
2	39 (2.7)	447 (30.5)	
3	54 (3.7)	674 (46.0)	
Chemotherapy			
−	115 (8.1)	1026 (72.4)	<0.05
+	16 (1.1)	260 (18.4)	
Endocrine therapy			
−	83 (5.9)	765 (54.0)	0.51
+	49 (3.5)	520 (36.7)	

*P* values are from Fisher’s exact tests except for grade, which is from *χ*^2^ tests.

High Na_v_1.5 protein expression was associated with a significant reduction in metastasis-free survival (HR 2.18; 95% CI 1.63–2.92; *P* < 0.001; log-rank test; Fig. 1F). This result was reflected in a significant reduction in overall survival (Supplementary Fig. 2A), cancer-specific survival (Supplementary Fig. 2B), disease-free survival (Supplementary Fig. 2C), and local recurrence-free survival (Supplementary Fig. 2D). When subdivided by receptor status, high Na_v_1.5 expression was associated with significantly reduced overall survival in ER+ (*P* < 0.05), but not HER2+ (*P* = 0.14) or TNBC patients (*P* = 0.53), although the sample sizes for HER2+ and TNBC patients were considerably smaller than for ER+ patients (Supplementary Fig. 2E–G).

The prognostic value of Na_v_1.5 protein expression was considered in a Cox proportional hazards model including tumour size, grade, and lymph node status as categorical variables. Na_v_1.5 expression was an independent predictor of survival alongside the other variables in this model (HR = 1.58; 95% CI 1.05–2.37, *P* < 0.05; Table 2). Finally, the correlation between Na_v_1.5 expression and other protein markers previously scored in the same breast cancer TMA was explored [25]. This analysis revealed that Na_v_1.5 expression was significantly positively correlated with several other invasion-related protein markers (Supplementary Table 1). In summary, high Na_v_1.5 protein expression is associated with worse prognosis in combined subtypes of breast cancer patients across a range of clinical measures, highlighting the proposed role of this ion channel in promoting invasion and metastasis.

**Table 2:** Cox multivariate analysis of cancer-specific survival.

Variable	Hazard ratio (95% CI)	*P* value
Tumour size		
≤20 mm	1	
>20 mm	1.16 (1.10–1.22)	<0.001
Grade		
1	1	
2	1.46 (1.01–2.11)	<0.05
3	2.62 (1.86–3.70)	<0.001
Lymph node status		
Negative	1	
Positive	1.65 (1.36–2.00)	<0.001
Na_v_1.5 expression		
Low	1	
High	1.58 (1.05–2.37)	<0.05

### Cells in patient breast cancer tissue exhibit voltage-sensitive inward and outward membrane currents

Much work has shown the potential for Na_v_1.5 to increase invasion and metastasis in cell culture models of breast and other epithelial cancers but until now, no electrophysiological recordings of Na_v_1.5 currents have been shown in tissues taken directly from cancer patients. To address this, we performed electrophysiological experiments to record membrane currents in fresh tissue samples from three breast tumours (Supplementary Table 2). In fresh tissue slices made from these patient tumour biopsies, there were few cellular areas and most of the slices were composed of connective tissue or fat. Thus, we took patch clamp recordings from pockets of cells within the connective tissue at the top surface of the slice. Adipocytes were identified based on their large size and avoided. Portions of each tumour were also dissociated and seeded onto coverslips to enable patch clamp recording from isolated cells.

**Fig. 2:**
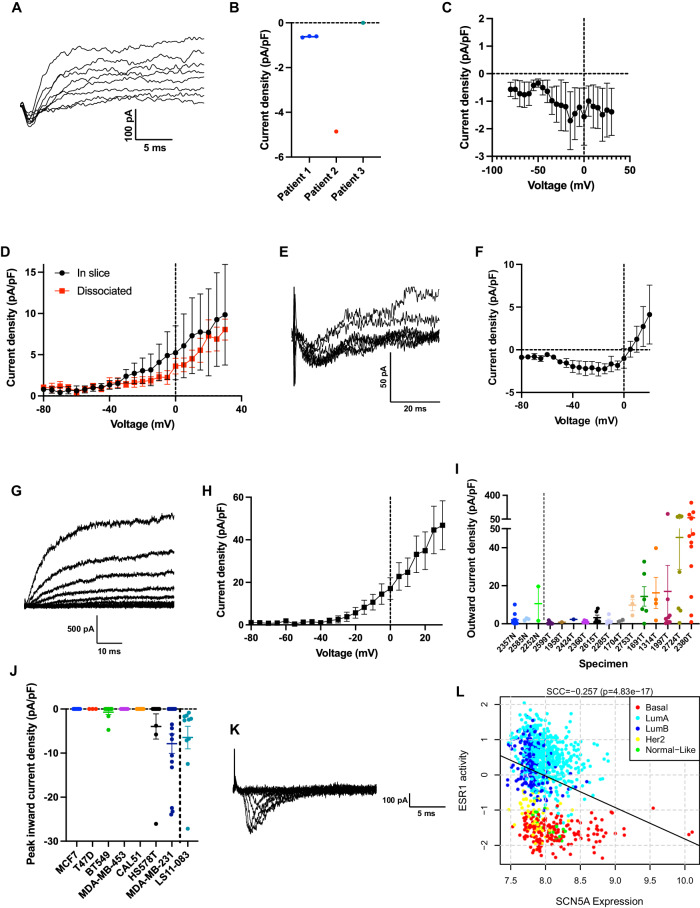
Characterisation of membrane currents in breast cancer tissue, dissociated primary cells, and cell lines. **A** Example inward and outward membrane currents recorded from a cell in a patient tumour slice, from a holding potential of −120 mV with 10 mV depolarising steps in the range −40 mV to +30 mV. **B** Peak inward current density, subdivided by patient tumour specimen. **C** Mean inward current–voltage relationship including cells from both patient tumour specimens (*n* = 4). **D** Mean outward current–voltage relationship from cells in patient tumour slices compared to cells which had been dissociated and plated onto glass coverslips (in slice: *n* = 10 cells from 3 patients; dissociated: *n* = 6 cells from 2 patients). There is no significant difference between in-slice and dissociated currents (*P* = 0.70; two-way ANOVA). **E** Example inward currents recorded from a dissociated mammary carcinoma cell, elicited by depolarising steps between −40 mV and −5 mV from a holding potential of −120 mV. **F** Mean inward current–voltage relationship from dissociated mammary carcinoma cell samples (*n* = 4 cells). **G** Example outward currents recorded from a dissociated mammary carcinoma cell, using a holding potential of −120 mV with 10 mV depolarising steps in the range −80 mV to +30 mV. **H** Mean outward current–voltage relationship from dissociated mammary carcinoma cells (*n* = 30 cells). **I** Outward current density at +30 mV of dissociated normal mammary epithelial and carcinoma cells, subdivided by patient sample. **J** Peak inward current density measured across a panel of breast cancer cell lines and cancer-associated fibroblasts (CAFs). **K** Example voltage-sensitive inward currents in an LS11-083 CAF. **L** Correlation of *SCN5A* mRNA (encoding Na_v_1.5) with estrogen receptor (ER) activity in the TCGA breast cancer dataset. Data are mean ± SEM.

Voltage clamp recording revealed that cells in tumour slices expressed both voltage-sensitive inward and outward currents (Fig. 2A). Small voltage-sensitive inward currents (characteristic of the type of inward current carried by Na_v_1.5 [26]) were found in two out of three patient specimens and in 4/17 recordings made in the tumour slices (Fig. 2B). The mean inward current–voltage relationship was noisy due to the small current density; however, it displayed activation at approximately −50 mV (Fig. 2C), consistent with Na_v_1.5 currents recorded from breast cancer cell lines [14, 26].

Comparisons were then made between the cells in tumour slices, and cells dissociated from the same tumours plated onto glass coverslips. The mean outward current–voltage relationship was similar between cells in slices and those dissociated onto coverslips (*P* = 0.70; two-way ANOVA; Fig. 2D). It was not possible to further characterise the inward currents due to the small number of cells exhibiting these currents. In summary, both outward and inward voltage-sensitive currents were detected in cells in breast cancer tissue slices taken directly from patients, although making such recordings was technically challenging due to limited availability and the fibrous composition of the tissue. Since the dissociation of tissue into isolated cells did not appear to affect the electrophysiological recordings, we moved on to recording from primary cell cultures.

### Primary breast epithelial and carcinoma cells exhibit voltage-sensitive inward and outward membrane currents

We recorded membrane currents from a total of 4 normal human mammary epithelial cell samples and 13 breast cancer samples enriched for carcinoma cells (Supplementary Table 3). Voltage-sensitive inward currents were present in 2 out of 13 breast cancer cell samples and none of 4 normal human mammary epithelial cell samples (Fig. 2E). The mean current–voltage relationship of the inward currents displayed activation at approximately −50 mV, again consistent with VGSC currents recorded from breast cancer cell lines (Fig. 2F) [14, 26]. Conversely, voltage-sensitive, non-inactivating outward currents were present in all normal human mammary epithelial cell samples tested and 10 out of 13 breast cancer cell samples (Fig. 2G–I). In summary, (i) non-inactivating voltage-gated outward currents were common in cultured normal human mammary epithelial cells and primary breast cancer cells whilst (ii) voltage-gated inward currents were rarely detectable, and only in malignant cells. The cell samples did not contain enough viable cells to allow further characterisation of the membrane currents or quantification of protein levels, however, patch clamping is a more sensitive measure of plasma membrane ion channel expression than western blot, given the rarity of these proteins.

### Inward currents are present in several triple-negative breast cancer cell lines and cancer-associated fibroblasts

To further explore the presence of VGSC currents in breast cancer, we set out to record from a panel of breast cancer cell lines and a breast tumour-derived cancer-associated fibroblast (CAF) cell line. Inward currents were present in MDA-MB-231 cells, consistent with previous reports [12, 14], and were also detected in Hs578T and BT549 cells (Fig. 2J). Notably, all three cell lines in which inward currents were detected are from TNBC. In addition, inward currents were present in the CAF line LS11-083, with activation at around −25 mV (Fig. 2K). Thus, we found functionally active VGSC currents in some TNBC and CAF cell lines, in broad agreement with several previous mRNA, protein, and electrophysiology studies [10–12, 14]. The presence of the inward currents in TNBC cell lines broadly agrees with the TMA data, where Na_v_1.5 expression was inversely correlated with ER and PR status (Table 1 and Supplementary Fig. 1). The inverse relationship between inward current and ER status also matches our previous electrophysiological observations in cell lines [12, 27]. Moreover, *SCN5A* (encoding for Na_v_1.5) expression was inversely correlated with ER activity in the TCGA breast cancer cohort, further supporting the possibility that VGSC expression/activity may be a feature of TNBC (*P* < 0.001; Fig. 2L).

### Na_v_1.5 regulates the expression of migration and invasion-promoting genes

In the TMA study, we showed that higher Na_v_1.5 protein expression correlated with increased metastasis and more invasive tumours (Fig. 1). In accordance with this, Na_v_1.5 activity has previously been shown to increase invasion and metastasis in preclinical models of breast cancer [11, 12, 14, 16–18, 24]. Because of these findings, we hypothesised that the knockdown of Na_v_1.5 would suppress the expression of invasion-related genes. To test this hypothesis, we next compared gene expression in six MDA-MB-231 xenograft tumours with six tumours in which *SCN5A* expression had been stably suppressed using shRNA [11]. We previously showed that Na_v_1.5 protein expression and Na^+^ current were ablated in these cells [11]. Principal component analysis showed that the *SCN5A* knockdown explained 25% of the variance and there was no relationship to mouse cage/block (Supplementary Fig. 3A, B).

**Fig. 3:**
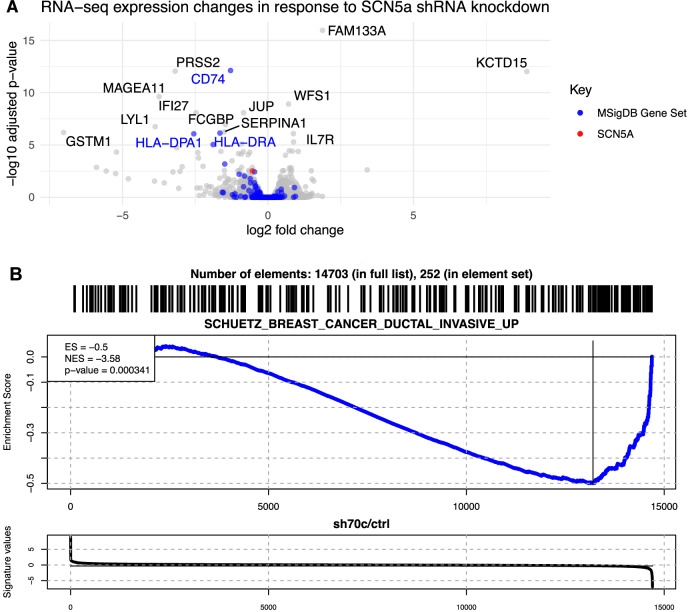
Altered human gene expression in Na_v_1.5 knockdown MDA-MB-231 xenograft tumours compared to control MDA-MB-231 tumours. **A** Volcano plot showing transcriptome changes in Na_v_1.5 knockdown tumours, with genes involved in invasion, and *SCN5A*, highlighted. **B** Gene set enrichment analysis (GSEA) of invasion-upregulated genes defined in the MSigDB gene set SCHUETZ_BREAST_CANCER_DUCTAL_INVASIVE_UP analysed in Na_v_1.5 knockdown vs. control tumours (*n* = 6 tumours in each condition).

The *SCN5A* shRNA tumours displayed 136 differentially expressed genes, compared to the control tumours (BH-adjusted *P* < 0.05; Fig. 3A). Gene ontology (GO) enrichment analysis for the ontologies ‘biological process’, ‘molecular function’, and ‘cellular compartment’ revealed several terms related to cellular migration and invasion, including homotypic cell–cell adhesion, regulation of actin filament-based process, as well as various lysosomal, endosomal, and membrane-related terms (Supplementary Fig. 4). We next performed gene set enrichment analysis (GSEA) to evaluate the relationship between *SCN5A* knockdown and invasion-promoting genes [28]. We used the MSigDB gene set SCHUETZ_BREAST_CANCER_DUCTAL_INVASIVE_UP which describes genes upregulated in invasive ductal carcinoma vs. ductal carcinoma in situ, a non-invasive type of breast tumour [29]. GSEA of the differential expression in *SCN5A* shRNA tumours showed a significant reduction in invasion transcriptional response (*P* < 0.001; Fig. 3B). There was also a smaller reduction in expression of invasion-downregulated genes (normalised enrichment score −2.35 vs. −3.58), although this was only significant at a reduced stringency (*P* < 0.05; Supplementary Fig. 3C). These results support the notion that Na_v_1.5 is a key driver of invasion in breast cancer cells.

### Na^+^ influx via Na_v_1.5 promotes glycolytic H^+^ production

**Fig. 4:**
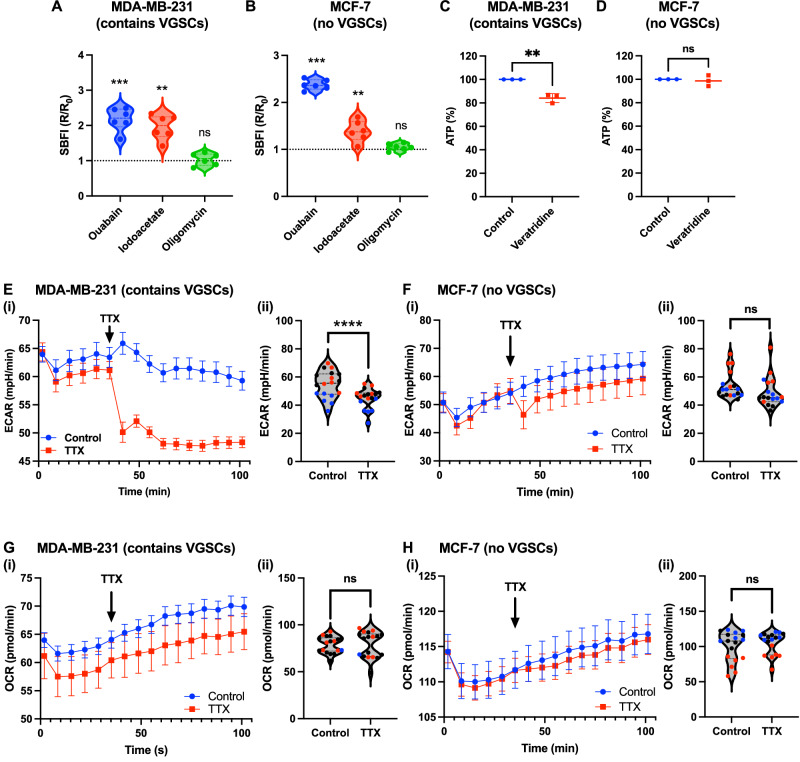
Effect of voltage-gated Na^+^ channel activity on glycolytic H^+^ production. **A** SBFI-AM fluorescence ratios of MDA-MB-231 cells after 6 h treatment with ouabain (300 nM), or 2 h treatment with sodium iodoacetate (2 mM) or oligomycin (1 µM). Data are normalised to the ratio in vehicle-treated cells (*n* = 6 experimental repeats; one-sample *t* tests). **B** SBFI-AM fluorescence ratios of MCF7 cells after 6 h treatment with ouabain (300 nM), or 2 h treatment with sodium iodoacetate (2 mM) or oligomycin (1 µM). Data are normalised to the ratio in vehicle-treated cells (*n* = 6 experimental repeats; one-sample *t* tests). **C** ATP content (normalised to control) of MDA-MB-231 cells after 90 min treatment in glucose-free physiological saline solution (PSS) ± veratridine (100 µM; *n* = 3 experimental repeats; *t* tests). **D** ATP content (normalised to control) of MCF7 cells after 90 min treatment in glucose-free PSS ± veratridine (100 µM; *n* = 3 experimental repeats; *t* tests). **E**(i) Representative measurements of the extracellular acidification rate (ECAR) of MDA-MB-231 cells. TTX was added to the treated cells at the indicated timepoint, to give a final concentration of 30 µM (*n* = 6 wells). **E**(ii) ECAR of MDA-MB-231 cells compared between control and TTX (30 µM) wells. **F**(i) Representative measurements of the ECAR of MCF7 cells. TTX was added to the treated cells at the indicated timepoint, to give a final concentration of 30 µM (*n* = 6 wells). **F**(ii) ECAR of MCF7 cells compared between control and TTX (30 µM) wells. **G**(i) Representative measurements of the oxygen consumption rate (OCR) of MDA-MB-231 cells. TTX was added to the treated cells at the indicated timepoint, to give a final concentration of 30 µM (*n* = 6 wells). **G**(ii) OCR of MDA-MB-231 cells compared between control and TTX (30 µM) wells. **H**(i) Representative measurements of the OCR of MCF7 cells. TTX was added to the treated cells at the indicated timepoint, to give a final concentration of 30 µM (*n* = 6 wells). **H**(ii) OCR of MCF7 cells compared between control and TTX (30 µM) wells. Each data point in (ii) represents the mean of the last 6 timepoints for each well (*n* = 3 experimental repeats each containing 6 wells; experimental repeats colour-coded black, red, blue). Data are mean ± SEM. ****P < 0.0001, ***P < 0.001, **P < 0.01, ns not significant (two-way ANOVA).

A common feature of many types of cancer cells is a tendency to shift their metabolism to aerobic glycolysis and therefore H^+^ production [30]. In MDA-MB-231 cells, Na_v_1.5 activity has been shown to increase H^+^ extrusion via NHE1, thus providing a lower extracellular pH (pH_e_) optimal for cysteine cathepsin-dependent extracellular matrix degradation and increased invasion [23, 24, 31]. However, the mechanistic link between Na_v_1.5 activity and NHE1-mediated H^+^ extrusion is unclear, given that Na^+^ influx through Na_v_1.5 would be expected to reduce the driving force for H^+^ export via NHE1. One possible explanation for the observed effect is that Na^+^ influx via Na_v_1.5 increases NKA activity to remove additional intracellular Na^+^ [32, 33] and maintain homoeostasis. NKA has been shown to use ATP predominantly derived from glycolysis in many tissues including breast cancer cells [34–36]. This dependence on glycolysis has been proposed to occur because it produces ATP close to the plasma membrane and the rate can quickly increase to cope with fluctuating demands from plasma membrane pumps [34]. Thus, we hypothesised that Na^+^ entry through Na_v_1.5 would lead to an increase in glycolytic rate and acidic metabolite production. To test this hypothesis, we first examined the source of ATP used by NKA in breast cancer cells. As expected, the NKA inhibitor ouabain (300 nM; 6 h incubation) increased [Na^+^]_i_, measured using the ratiometric Na^+^ indicator SBFI-AM, by 2.2-fold in MDA-MB-231 cells (*P* < 0.001; *n* = 6; one-sample *t* test; Fig. 4A) and by 2.0-fold in MCF7 cells (*P* < 0.001; *n* = 6; one-sample *t* test; Fig. 4B). This indicated that a rise in [Na^+^]_i_ could be used as a proxy for NKA inhibition. Interestingly, complete inhibition of mitochondrial respiration using the ATP synthase inhibitor oligomycin (1 µM; 2 h) did not alter [Na^+^]_i_ in MDA-MB-231 (*P* = 0.62; *n* = 6; one-sample *t* test; Fig. 4A) or MCF7 cells (*P* = 0.14; *n* = 6; one-sample *t* test; Fig. 4B). However, inhibition of glycolysis with the GAPDH inhibitor sodium iodoacetate (2 mM; 2 h) significantly increased [Na^+^]_i_ in both cell lines (*P* < 0.01 for both cell lines; *n* = 6; one-sample *t* test; Fig. 4A, B). A second glycolysis inhibitor, 3-bromopyruvate [37], also increased [Na^+^]_i_ in MDA-MB-231 cells (Supplementary Fig. 5). These data suggest that NKA was able to maintain a steady [Na^+^]_i_ in the absence of mitochondrial respiration but not in the absence of glycolysis. Oligomycin had no effect on the viability of either cell line (*P* = 0.74 for MDA-MB-231 and *P* = 0.84 for MCF7; *n* = 3; one-sample *t* tests), whereas sodium iodoacetate significantly reduced viability in both cell lines (*P* < 0.01 for MDA-MB-231 and *P* < 0.05 for MCF7; *n* = 3; one-sample *t* tests; Supplementary Fig. 6A, B). Thus, these data suggest that in both MDA-MB-231 and MCF7 cells, NKA activity requires ATP derived from glycolysis to export Na^+^, and it can function without mitochondrial respiration.

To test the hypothesis that Na_v_1.5 activity increases ATP demand, we next measured cellular ATP content using the CellTiter-Glo assay following incubation in a glucose-free physiological saline solution (PSS). Treatment of MDA-MB-231 cells with the VGSC ‘opener’ veratridine [26] (100 µM; 90 min) significantly reduced ATP content in the absence of glucose (*P* < 0.01; *n* = 3; *t* test; Fig. 4C). In contrast, veratridine did not affect the ATP content of MCF7 cells, which lack functional Na_v_1.5 channels (*P* = 0.68; *n* = 3; *t* test; Fig. 4D). Treatment with veratridine did not affect the viability of either cell line (Supplementary Fig. 6C, D). Together with the above findings, these results suggest that Na_v_1.5 activity increases glycolytic ATP demand from NKA.

Next, we used a Seahorse XFe96 analyzer to test the hypothesis that Na^+^ influx through Na_v_1.5 increases the rate of glycolysis via increasing ATP demand from NKA. The effects of the VGSC inhibitor tetrodotoxin citrate (TTX, 30 µM) on the extracellular acidification rate (ECAR; a measure of glycolysis) and oxygen consumption rate (OCR; a measure of mitochondrial respiration) were compared between MDA-MB-231 cells (which express Na_v_1.5, but no other functional VGSC subtypes [11, 12, 23, 26, 31]) and MCF7 cells (which do not display these currents; Fig. 2J; [12, 14]). In control experiments, the addition of TTX to wells containing medium without cells transiently reduced the measured ECAR, which then returned to baseline levels within 15 min (Supplementary Fig. 7). Therefore, measurements on cells ± TTX were compared after this period. The addition of TTX (30 µM) to MDA-MB-231 cells caused a rapid and sustained reduction in ECAR (*P* < 0.0001; *n* = 3 experimental repeats containing 6 wells each; two-way ANOVA; Fig. 4E). However, TTX had no significant effect on the ECAR of MCF7 cells, which do not express functional Na_v_1.5 channels [38] (*P* = 0.07; *n* = 3 experimental repeats containing 6 wells each; two-way ANOVA; Fig. 4F). In contrast, OCR was unaffected by TTX in both cell lines (*P* = 0.99 and 0.11 for MDA-MB-231 and MCF7, respectively; *n* = 3 experimental repeats containing 6 wells each; two-way ANOVA; Fig. 4G, H). Together, these data are consistent with Na^+^ influx via Na_v_1.5 increasing the rate of glycolysis but not mitochondrial respiration. This result can therefore explain the established link between Na_v_1.5 activity and NHE1-induced extracellular acidification which promotes invasion [31]. It also highlights a novel link between Na^+^ homoeostasis and altered metabolism in cancer cells.

### Extracellular pH is lower towards the periphery of xenograft tumours and low pH correlates with high cellularity and proliferation

**Fig. 5:**
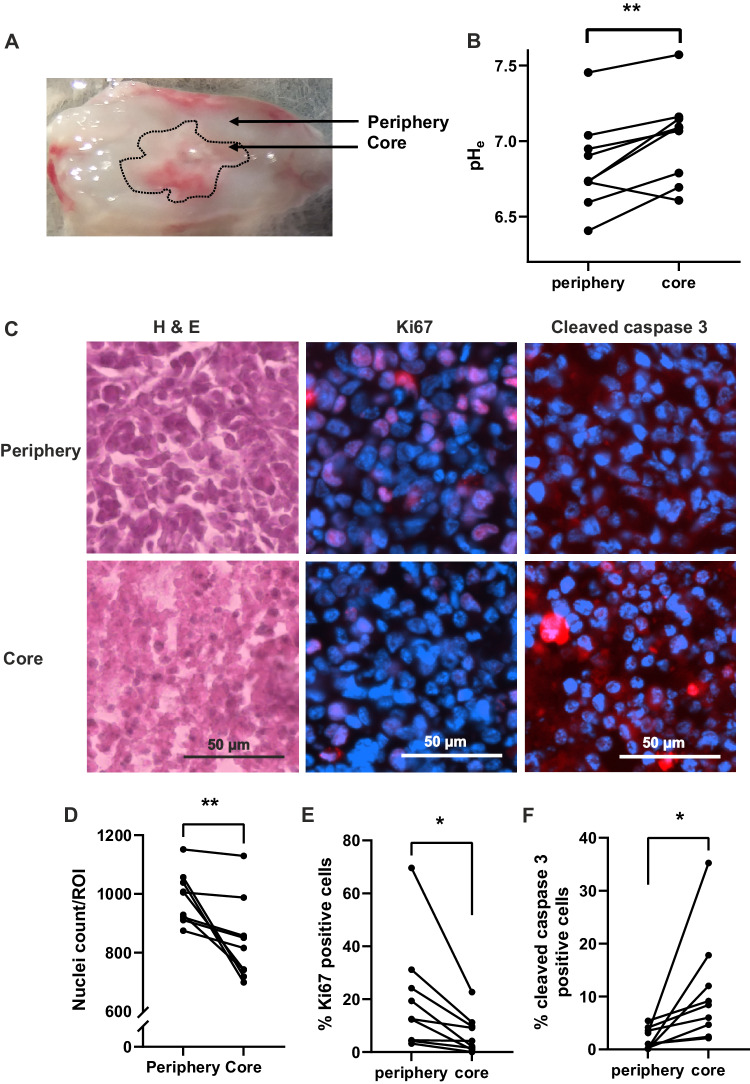
Altered extracellular pH in tissue slices from MDA-MB-231 xenograft tumours. **A** Photograph of a tumour slice showing the difference in appearance between the translucent periphery and opaque core. **B** Tumour slice extracellular pH (pH_e_) comparing core and peripheral regions in each slice; *n* = 9 tumours (one slice from each). **C** Examples of peripheral and core regions with H&E staining (left), Ki67 (red, middle), and cleaved caspase 3 (red, right) with DAPI (blue). **D** Cellularity in the peripheral and core regions of each section, based on DAPI nuclear count (*n* = 9 tumours). **E** Ki67-positive cells (%) in peripheral and core regions (*n* = 9 tumours). **F** Cleaved caspase 3-positive cells (%) in peripheral and core regions (*n* = 9 tumours). Data are mean ± SEM; *P < 0.05, **P < 0.01 (paired *t* tests).

The tumour microenvironment is reported to be acidic [39], with a low intratumoural pH facilitating various metastatic hallmarks including ECM degradation and invasion. Classically, this low pH has been thought to be due to hypoxia in the poorly perfused areas of the tumour. Here we show evidence that low pH_e_ can instead be associated with highly proliferative, peripheral areas of the tumour where it would be expected that there is increased metabolic activity. This would be consistent with areas of high glycolytic activity, and potentially high NKA activity. We, therefore, next assessed the pH_e_ of MDA-MB-231 xenograft tumours using pH-sensitive microelectrodes. Measurements were recorded in several locations on the top surface of tissue slices directly prepared from xenograft tumours, alternately from the opaque core of the tumour, and from more translucent periphery (Fig. 5A). The mean overall pH_e_ was 6.9 ± 0.1, which is significantly lower than pH 7.4 normally found in the extracellular fluid of healthy tissue (*P* = 0.001; *n* = 9; one-sample *t* test) [39]. The mean pH_e_ in the core was 7.0 ± 0.1; in the periphery, it was significantly lower, at 6.8 ± 0.1 (*P* < 0.01; *n* = 9; paired *t* test; Fig. 5B). The differences between core and periphery were investigated in more detail using immunohistochemistry (Fig. 5C). Cellularity (mean nuclear count per ROI) was significantly higher in the periphery compared to the core (988 ± 29 vs. 838 ± 47; *P* < 0.01; *n* = 9 tumours; paired *t* test; Fig. 5D). Similarly, proliferation, as measured by Ki67-positive nuclei, was significantly higher in the periphery than the core (20.1 ± 7.0% in the periphery vs. 6.9 ± 2.4% in core; *P* < 0.05; *n* = 9 tumours; paired *t* test; Fig. 5E). Conversely, apoptosis, as measured by cleaved caspase 3 positivity, was significantly higher in the core compared to the periphery (10.9 ± 3.5% in core vs. 2.1 ± 0.7% in periphery; *P* < 0.05; *n* = 9 tumours; paired *t* test; Fig. 5F). Taken together, these results suggest that in this model the pH_e_ is lower in peripheral regions with high cellularity which are proliferating rapidly, and higher towards the core of the tumours, where there is more apoptosis.

### Persistent Na_v_1.5 current is increased by low extracellular pH

**Fig. 6:**
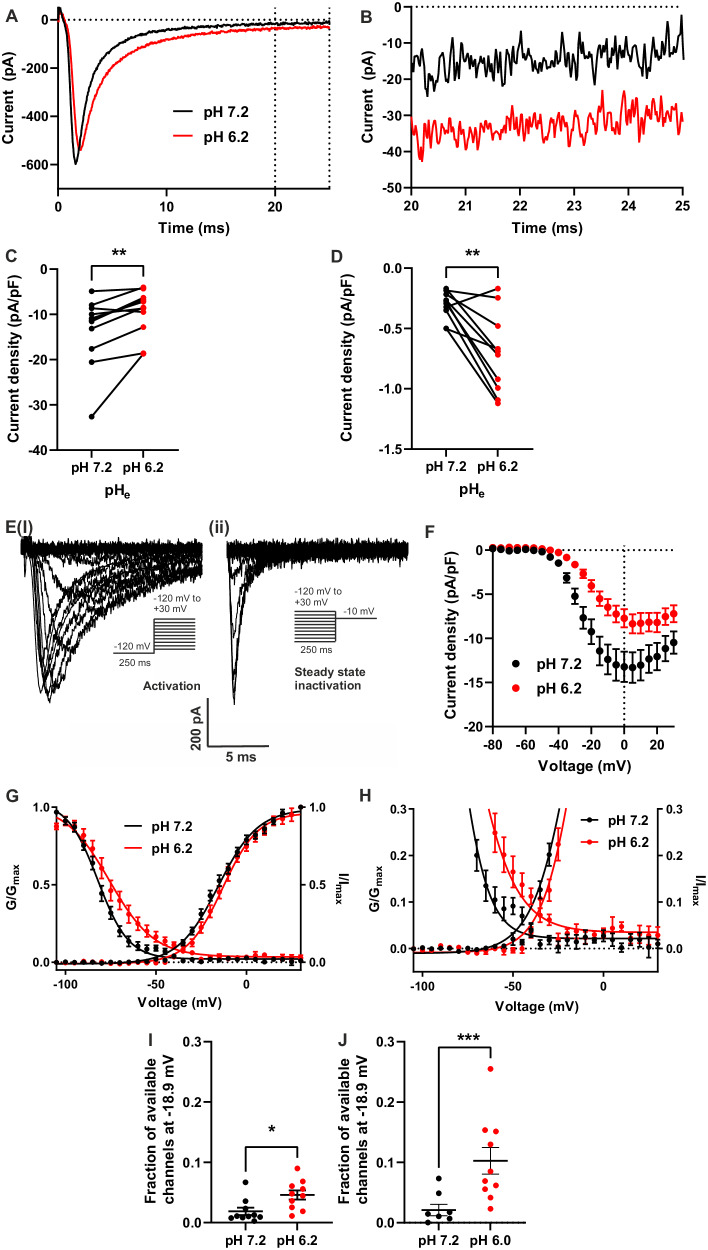
Effect of low pH_e_ on Na^+^ current in MDA-MB-231 cells. **A** Example Na^+^ currents elicited by depolarisation to 0 mV from a holding voltage of −120 mV. **B** Data from (**A**) expanded between 20 and 25 ms after depolarisation. **C** Peak Na^+^ current density (*P* < 0.01; *n* = 11; Wilcoxon matched pairs test). **D** Mean persistent Na^+^ current density measured between 20 and 25 ms after depolarisation (*P* < 0.01; *n* = 10; paired *t* test). **E**(i) Example family of Na^+^ currents generated by the activation voltage clamp protocol (inset). **E**(ii) Example family of Na^+^ currents generated by the steady-state inactivation voltage clamp protocol (inset). **F** Current density–voltage relationship (*n* = 17). **G** Overlay of activation and inactivation curves at pH 7.2 and 6.2 (*n* = 10 cells with the largest currents). **H** Expanded data from (**G**) showing the window current. **I** Fraction of channels available at the reported resting membrane potential of −18.9 mV at pH 7.2 and 6.2 (*n* = 10 cells with the largest currents; *t* test). **J** Fraction of channels available at the reported resting membrane potential of −18.9 mV, at pH 7.2 and 6.0 (*P* < 0.001; *n* = 8 cells; *t* test). Data are mean ± SEM. ***P < 0.001, **P < 0.01, *P < 0.05.

Given that the tumour pH_e_ is acidic in vivo, we next assessed the effect of pH alteration on Na_v_1.5 activity in MDA-MB-231 cells using patch clamp recording. A pH_e_ of 7.2 was compared with a pH_e_ of 6.2 since the pH_e_ of solid tumours has been reported to approach this level of acidity [40]. Lowering pH_e_ to 6.2 reduced the transient Na^+^ current from −13.5 ± 2.3 pA/pF to −9.6 ± 1.5 pA/pF (*P* < 0.01; *n* = 11; Wilcoxon matched pairs test; Fig. 6A, C). In contrast, the persistent Na^+^ current, measured at 20–25 ms after depolarisation, was increased by acidification to pH_e_ 6.2, from −0.31 ± 0.04 pA/pF to −0.71 ± 0.11 pA/pF (*P* < 0.01; *n* = 10; paired *t* test; Fig. 6B, D). Analysis of the voltage dependence of activation and steady-state inactivation (Fig. 6E–G) revealed that the slope factor (*k*) and voltage at half activation (*V*½) did not significantly change when the pH_e_ was reduced from 7.2 to 6.2 (*P* = 0.077 and 0.087, respectively; *n* = 10; paired *t* tests; Table 3). However, the *V*½ was significantly depolarised at pH_e_ 6.2, from −80.4 ± 1.4 mV to −73.3 ± 2.8 mV (*P* < 0.01; *n* = 10; paired *t* test; Table 3) and the *k* for inactivation was also significantly changed, from −8.4 ± 0.8 mV to −11.9 ± 0.9 mV (*P* < 0.01; *n* = 10; paired *t* test; Table 3). This depolarising shift in steady-state inactivation thus increased the size of the window current (Fig. 6G, H). Indeed, at the reported resting *V*_m_ of MDA-MB-231 cells, −18.9 mV [12], reducing the pH_e_ from 7.2 to 6.2 more than doubled channel availability from 1.9 ± 0.6% to 4.9 ± 0.7% of maximum (*P* < 0.05; *n* = 10; paired *t* test; Fig. 6I). When pH_e_ was reduced further to 6.0, the effect on channel availability was even greater, increasing nearly fivefold from 2.1 ± 0.9% at pH_e_ 7.2 to 10.3 ± 2.2% of maximum at pH_e_ 6.0 (*P* < 0.001, *n* = 8, paired *t* test; Fig. 6J; Supplementary Fig. 8).

**Table 3:** Effect of reduced pH_e_ on VGSC Na^+^ current parameters.

Parameter	pH 7.2	pH 6.2	*P*	*n*
Peak current density (pA/pF)	−13.5 ± 2.3	−9.6 ± 1.5	0.006	11
Persistent current density (pA/pF)	−0.31 ± 0.04	−0.71 ± 0.11	0.006	10
Activation *V*½ (mV)	−15.2 ± 2.0	−12.9 ± 1.8	0.087	10
Activation *k* (mV)	10.7 ± 0.5	9.5 ± 0.4	0.077	10
Inactivation *V*½ (mV)	−80.4 ± 1.4	−73.3 ± 2.8	0.003	10
Inactivation *k* (mV)	−8.4 ± 0.8	−11.9 ± 0.9	0.005	10

The holding potential was −120 mV. Results are mean ± SEM. Statistical comparisons were made with paired *t* tests on non-normalised data.

*V½* half (in)activation voltage, *k* slope factor for (in)activation.

Cancer cells have been shown to have an inverted pH ratio across the plasma membrane, so the exterior of the cell is more acidic but the intracellular fluid has a higher pH than normal cells [39]. For this reason, it was important to explore whether the pH_e_-dependent electrophysiological changes were mediated by a change in intracellular pH (pH_i_). To do this we assessed the effect of pH_e_ on pH_i_ using the ratiometric fluorescent pH indicator BCECF-AM to measure pH_i_ following incubation in different pH_e_. Lowering pH_e_ from 7.2 to 6.0 led to intracellular acidification to pH_i_ 6.3 ± 0.1 (Supplementary Fig. 9A, B). However, altering pH_i_ from 7.2 to 7.6 (empirically the maximum range of intracellular patch pipette solution pH which still allowed the formation of giga-Ohm seals onto MDA-MB-231 cells) had no effect on transient or persistent Na^+^ current or voltage dependence of activation or steady-state inactivation (Supplementary Fig. 9C–G). In summary, Na^+^ entry into breast cancer cells through Na_v_1.5 is increased in acidic pH_e_ but is not sensitive to changes in pH_i_ under the range of pH_i_ tested. These data suggest that areas of the tumour with lower pH_e_ would have increased persistent Na^+^ current into breast cancer cells at steady state. This additional Na^+^ influx would either lead to a slow and continuous increase in intracellular [Na^+^], resulting in cell death (the opposite of what we observed in the more acidic parts of the tumour), or it would lead to increased NKA activity in the more acidic parts of the tumour, to maintain a stable intracellular [Na^+^] and maintain cell viability.

### Prediction of Na_v_1.5-dependent extracellular acidification rate

The expected ECAR due to VGSC activity can be calculated if it is assumed that the persistent Na^+^ current into cells through VGSCs is counteracted by the activity of NKA to maintain a stable [Na^+^]_i_. The other assumptions used in this calculation are that NKA pumps three Na^+^ ions out of the cell per cycle in which it hydrolyses one molecule of ATP [41]. Glycolytic production of lactic acid produces two molecules of ATP and two molecules of lactate per glucose. The production of H^+^ by this reaction coupled to the ATPase hydrolysis of ATP to ADP generates two H^+^ per glucose [42]. There is, therefore, a net production of one H^+^ per ATP molecule generated by glycolytic fermentation to produce lactate. Using these assumptions, we calculated the ECAR due to Na_v_1.5 activity to be 1.3 mpH/min (full calculations delineated in Supplementary Materials). This predicted ECAR is within an order of magnitude of the measured change in ECAR due to TTX inhibition of Na_v_1.5 (9.8 ± 1.7 mpH/min; Fig. 4D). Thus, our model can explain how Na_v_1.5 activity can increase H^+^ extrusion through NHE1, considering experimental variability in determination of ECAR and persistent Na^+^ current, and estimation of pH_e_ during the Seahorse assay.

### Protein–protein interactions of NHE1

NHE1 is the pH regulator which has been implicated as most important in Na_v_1.5-dependent extracellular acidification and protein–protein interactions have been suggested to play a significant role in regulating NHE1 activity, including by Na_v_1.5 [23, 43, 44]. We therefore looked for other protein interactions of NHE1. Using the STRING database (v11.5) we searched for the top 50 likely protein interactions of NHE1, only considering the proteins sharing a physical complex. This identified several subunits of NKA as likely binding partners of NHE1 (Supplementary Fig. 10). An interaction between NKA and NHE1 further supports a model in which NKA is an intermediate step by which Na^+^ influx through channels such as Na_v_1.5 can then alter NHE1 activity.

**Fig. 7:**
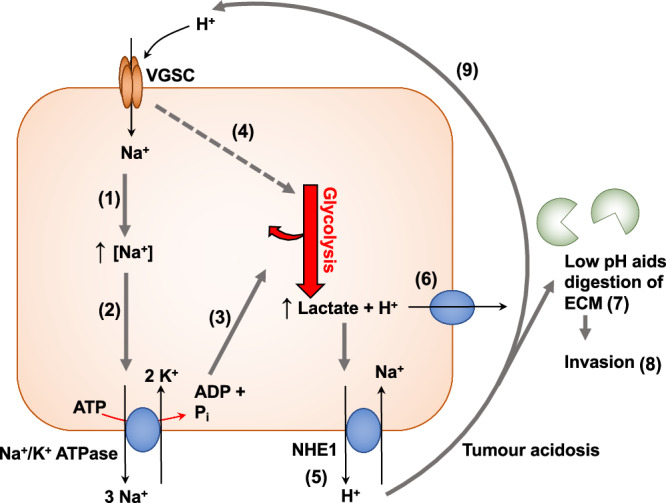
Mechanism for Na_v_1.5-mediated cellular invasion. Elevated steady-state Na^+^ entry through Na_v_1.5 leads to increased NKA activity. The increased ATP demand is satisfied by glycolysis, increasing H^+^ production and extrusion through pH regulators such as CAIX and NHE1. Extracellular acidification, in turn, increases persistent Na^+^ entry via Na_v_1.5, leading to a positive feedback loop linking Na_v_1.5, Na^+^ entry, increased H^+^ production via glycolysis, and extracellular acidification. This acidification results in ECM degradation and increased invasion. The evidence supporting this mechanism is as follows (corresponding to the numbering on the figure): (1) VGSC activity increases [Na^+^]_i_ in cancer cells [26, 87, 88]. (2) Elevated [Na^+^]_i_ promotes NKA activity, increasing ATP demand [32, 33] (Fig. 4). (3) ATP demand from NKA is satisfied through increased glycolysis [34–36, 59, 60, 89]. (4) Na_v_1.5 activity increases glycolysis (Fig. 4). (5) Na_v_1.5 activity increases H^+^ export via NHE1 [23, 31]. (6) Glycolysis increases H^+^ export via multiple extrusion routes [30, 62]. (7) Na_v_1.5 and low pH promote digestion of the ECM [24, 30]. (8) Na_v_1.5 increases invasion [7, 11, 12, 14, 16, 17, 31, 90]. (9) H^+^ increases persistent Na^+^ current through Na_v_1.5 [91] (Fig. 6).

In summary, as well as identifying a mechanism by which Na_v_1.5 may increase tumour acidification via an enhanced rate of glycolysis, our data suggest that the acidic tumour microenvironment could increase Na^+^ influx via Na_v_1.5. Together, these findings suggest that there is a positive feedback loop in breast cancer cells, whereby Na^+^ influx and H^+^ release into the tumour microenvironment could synergise to promote invasion and metastasis (Fig. 7).

## Discussion

In this study on breast cancer, we show that upregulation of Na_v_1.5 protein expression is positively associated with metastasis and reduced cancer-specific survival. We also identify a novel activity-dependent positive feedback role for this channel which results in increased metabolic activity and extracellular acidification. We have thus provided an integrated mechanism by which Na_v_1.5 can promote metastatic dissemination. We show specifically that acidity of the tumour microenvironment, particularly in the invasive periphery of the tumour, enhances persistent Na^+^ current through Na_v_1.5 into breast cancer cells, and this, in turn, promotes glycolytic metabolic activity. We, therefore, propose that Na_v_1.5 is a key regulator of the ionic tumour microenvironment, facilitating local invasion during the early stage of metastasis.

### Clinical significance of Na_v_1.5 expression in breast cancer

Our findings support, for the first time, a pro-metastatic role for Na_v_1.5 in the clinical setting. We show that Na_v_1.5 protein expression correlates with lymph node positivity, increased metastasis, higher tumour grade, and consequently reduced survival. In addition, the prognostic potential of Na_v_1.5 expression is independent of, but of comparable importance to, lymph node status, tumour grade, and tumour size. Although there have been several previous reports indicating that VGSCs are likely to be important prognostic indicators in breast cancer [10–12], no study has previously examined Na_v_1.5 protein expression in a large cohort of breast cancer patients. Previously, VGSC protein expression in breast cancer has mostly been extrapolated from experiments in cell lines with differing metastatic potential [12, 14] and small cohorts of patients [11–13]. Interestingly, we found that Na_v_1.5 expression was negatively associated with ER status, and positively correlated with HER2 status, but not with TNBC status. Together, these findings suggest a possible functional linkage between ER, HER2, and Na_v_1.5 expression. In agreement with this notion, ER+ MCF7 cells have low VGSC expression [12], and silencing ER in MCF7 cells increases Na^+^ current and VGSC-dependent invasion [27]. In addition, various tyrosine kinase receptors closely related to HER2, including EGFR, have been shown to regulate VGSC expression in carcinoma cells [45]. Further work is required to determine how these receptors interact with Na_v_1.5 in breast cancer cells.

Na_v_1.5 protein expression does not necessarily result in active channels at the plasma membrane. Therefore, for the first time, we attempted to investigate functional channel activity at the plasma membrane using whole-cell patch clamp recording. Although non-inactivating outward currents were widespread, small inward currents, indicative of Na_v_1.5 activity, were rarer and harder to detect. This result is surprising given the high proportion of Na_v_1.5-positive cells in the TMA. This apparent contradiction may be explained if a large proportion of the channels are present on intracellular membranes. Another possibility is that the tissue slice, dissociation, and cell culture conditions resulted in the transport of channels away from the plasma membrane, as has been shown for K_Ca_3.1 [46]. Indeed, the cancerous tissues and primary cultures contained very few cells viable enough for electrophysiological recording. A few studies have reported the presence of Na^+^ currents carried by VGSCs in cells dissociated from mesothelioma and cervical tumour tissue, although the latter had been maintained in long-term culture [47–49]. A recent study also demonstrated Na^+^ currents in primary colorectal carcinoma cells [43]. To our knowledge, ours is the first report of VGSC currents in breast cancer tissue or primary cells from patients, however, more work is required to refine the procedures for electrophysiological recordings using such clinical material, for example, by employing high-throughput patch clamp approaches [50, 51].

### Na_v_1.5-induced extracellular acidification – a positive feedback mechanism promoting invasion

Our unexpected finding that the proliferating and invasive tumour periphery was more acidic than the hypoxic core agrees with a number of other studies [39, 52–55]. Low extracellular pH caused by Na_v_1.5 activity promotes invasion by increasing the activity of low pH-dependent enzymes that degrade the ECM, such as cysteine cathepsins [24, 31], so it makes sense that the invading edge of a tumour should have a low pH_e_. We found that low pH_e_, as found in tumours, increases the persistent Na^+^ current through Na_v_1.5 in breast cancer cells, thereby promoting Na^+^ influx. Greater Na^+^ influx into cancer cells in tumour regions of lower pH_e_ may be partially responsible for the heterogeneity of apparent tumour [Na^+^] as measured by ^23^Na-MRI [56, 57].

Increased Na_v_1.5-mediated Na^+^ influx into cancer cells would be expected to promote the activity of NKA [58], and our data show that Na_v_1.5 activity increases ATP consumption. There is substantial evidence that NKA utilises glycolysis as its main ATP source [21, 34, 59, 60]. NKA activity, and consequent glycolytic metabolism, would therefore increase the rate of H^+^ production. In agreement with this paradigm, we found that Na_v_1.5 activity in breast cancer cells increased glycolysis, as measured by extracellular H^+^ production, without affecting oxidative phosphorylation. Increasing [Na^+^]_i_, via the ionophore gramicidin, has previously been shown to potentiate the rate of glycolysis in breast cancer cells, whereas the NKA inhibitor ouabain decreased H^+^ production [34]. These findings echo those where glycolysis was found to be the ATP source for another plasma membrane ion pump, the plasma membrane Ca^2+^ ATPase in pancreatic cancer cells [37, 61]. We found that inhibition of glycolysis induced a large increase in [Na^+^]_i_ similar to that caused by ouabain, and also rapidly led to cell death.

Elevated steady-state Na^+^ entry via persistent current through Na_v_1.5, leading to increased glycolysis to power NKA, would in turn, be expected to increase H^+^ extrusion through various pH regulators, e.g. carbonic anhydrase IX (CAIX), sodium-proton exchangers (NHEs), sodium-bicarbonate cotransporters, monocarboxylate transporters, and H^+^-ATPases [30, 62]. Reciprocally, the reduction in pH_e_ serves to increase persistent Na^+^ entry into breast cancer cells via Na_v_1.5. Together, these mechanisms would lead to a positive feedback loop linking Na_v_1.5, Na^+^ entry, increased H^+^ production via glycolysis, and extracellular acidification (Fig. 7). This model fits with previous studies showing that Na_v_1.5 activity increases H^+^ extrusion in breast cancer cells, leading to ECM degradation and increased invasion [23, 31]. It would also explain how Na^+^ influx via Na_v_1.5 increases H^+^ efflux via NHE1, despite an apparently wasteful collapse of the inward Na^+^ gradient that powers NHE1-mediated extrusion of H^+^ [7]. Given our evidence that extracellular acidification in breast tumours occurs particularly in the highly proliferative peripheral region, and that the persistent Na^+^ current through Na_v_1.5 is larger in acidic conditions, more Na^+^ would be likely to enter breast cancer cells at the invading edges of the tumour. This feed-forward mechanism would provide a self-sustaining supply of H^+^ to the extracellular space, thus increasing the activity of low pH-dependent proteases to drive invasion [24, 31].

## Conclusion

Here, we have shown that Na_v_1.5 is associated with poor prognosis and increased metastasis in breast cancer. Since Na_v_1.5 is a negative prognostic indicator, and its expression increases tumour growth and metastasis in preclinical models [11], it is a promising target for drug repurposing and discovery [10, 63, 64]. In agreement with this notion, we recently showed that exposure to certain persistent Na^+^ current-inhibiting Class 1c and 1d antiarrhythmic drugs is associated with significantly improved cancer-specific survival [65]. In addition, VGSC-inhibiting drugs have been shown to decrease tumour growth and metastasis in murine breast cancer models [17, 18]. Furthermore, a recent clinical trial has shown that presurgical peritumoral treatment with lidocaine significantly improves disease-free and overall survival in women with early breast cancer [19]. In conclusion, our results reveal a positive feedback mechanism by which Na^+^ influx through Na_v_1.5 promotes glycolytic H^+^ production to increase invasive capacity and drive breast cancer metastasis. This novel mechanism, together with the emerging clinical data, underscores the value of Na_v_1.5 as a prognostic marker and potential anti-metastatic therapeutic target. Based on this work, safe, orally available VGSC inhibitors, e.g. the persistent current blocking antianginal ranolazine [66], could be repurposed for use in early breast cancer patients at high risk of relapse, for example, to suppress invasion in the pre- and peri-operative period, and so prevent metastasis. Such an intervention may be particularly useful during surgery to treat inflammation and/or ‘showering’ with invasive cancer cells when the tumour is removed, as has been discussed elsewhere [66, 67]. In addition, systemic therapy with an Na_v_1.5 inhibitor may be valuable in patients with metastasis at diagnosis, thus suppressing further spread. Future work should be directed at establishing the effectiveness of VGSC inhibitors as metastasis suppressors, whilst also evaluating suitable physiological biomarkers of response.

## Materials and methods

### Breast cancer cell lines

MDA-MD-231, MCF7, T47D, MDA-MB-453, CAL51, BT549, and Hs578T cells were cultured in Dulbecco’s modified eagle medium (DMEM) supplemented with 5% foetal bovine serum (FBS) and 4 mM l-glutamine [68]. MDA-MB-231 and MCF7 cells were from M Djamgoz, Imperial College London. T47D, MDA-MB-453, CAL51, BT549, and Hs578T cells were from C Bakal, Institute of Cancer Research. Molecular identity was confirmed by short tandem repeat analysis [69]. MDA-MB-231 cells stably expressing shRNA targeting *SCN5A* were maintained in a medium containing G418 (400 μg/ml) [11]. LS11-083 hTERT-immortalised primary breast cancer-associated fibroblast cells (from V Speirs, University of Aberdeen) were also cultured in DMEM with 5% FBS and 4 mM l-glutamine [70]. Cultures were confirmed to be *Mycoplasma*-free using the 4′,6-diamidino-2-phenylindole (DAPI) method [71].

### Orthotopic xenograft breast cancer model and tissue slice preparation

*Rag2*^−/−^ *Il2rg*^−/−^ mice were bred in-house and females over the age of 6 weeks were used for tumour implantation. A suspension of 1 × 10^6^ MDA-MB-231 cells in Matrigel (Corning; 50% v/v in phosphate-buffered saline (PBS)) was implanted into the left inguinal mammary fat pad of each animal whilst under isoflurane anaesthesia. Mice were weighed and their body condition and tumour size were checked at least every 2 days. Tumours were measured using callipers and the tumour volume was calculated using the modified ellipsoidal formula, volume = ½(length × width^2^). Mice were euthanized after ~4 weeks. Tumours were dissected immediately after euthanasia and sliced in ice-cold PBS using a Campden 5100MZ vibratome to a thickness of 500 μm for pH-sensitive microelectrode recording.

### Human breast cancer tissue and primary cells

Biopsy samples from four patient breast tumours that were excess to pathology requirements were acquired via the Breast Cancer Now Tissue Bank (BCNTB). The samples were transported in culture medium on ice and arrived within 24 h of surgical resection. Fresh tissue slices (250 μm thick) were cut in ice-cold PBS using a vibratome (Campden 5100MZ). For isolation of single cells from the tumour tissue, fragments were cut and then dissociated using a MACS Tumour Dissociation Kit (Miltenyi Biotec). Primary cultures of breast cancer cells, enriched for carcinoma cells, and primary cultures of purified normal breast epithelial cells were also acquired via the BCNTB. Human primary cells and tumour slices were cultured in DMEM:F12 with 1 ml/100 ml penicillin/streptomycin, 2.5 μg/ml Fungizone, 10% FBS, 0.5 μg/ml hydrocortisone, 10 μg/ml apo-transferrin, 10 ng/ml human EGF and 5 μg/ml insulin. The cells were cultured on collagen-coated glass coverslips in plastic dishes.

### Whole-cell patch clamp recording

The whole-cell patch clamp technique was used to record cell membrane currents from cells grown on glass coverslips [72]. Filamented borosilicate capillary tubes were pulled and fire-polished to a resistance of ~5 MΩ for recording from cell lines and ~10 MΩ for recording from primary cells. The extracellular PSS contained (in mM) NaCl 144, KCl 5.4, MgCl_2_ 1, CaCl_2_ 2.5, HEPES 5, d-glucose 5.6, and was adjusted to pH 7.2 (unless otherwise stated) using NaOH. The intracellular recording solution for measuring Na^+^ currents contained (in mM) NaCl 5, CsCl 145, MgCl_2_ 2, CaCl_2_ 1, HEPES 10, EGTA 11 and was adjusted to pH 7.4 (unless otherwise stated) using CsOH [72]. The intracellular recording solution for measuring K^+^ currents contained (in mM) NaCl 5, KCl 145, MgCl_2_ 2, CaCl_2_ 1, HEPES 10, EGTA 11 and was adjusted to pH 7.4 using KOH. Recordings were made using a MultiClamp 700B amplifier (Molecular Devices). Currents were digitised using a Digidata 1440A interface (Molecular Devices), low-pass filtered at 10 kHz, sampled at 50 kHz, and analysed using pCLAMP 10.7 software (Molecular Devices). For the detection of small currents in primary cells, patient tumour slices and a panel of cell lines, signals were post-filtered at 1 kHz. For examination of pH dependency in MDA-MB-231 cells, currents were noise-corrected by subtracting half the peak-to-peak noise measured during the 10 ms period before depolarisation [72]. Series resistance was compensated by 40–60% and linear components of leak were subtracted using a P/6 protocol [73]. Cells were clamped at a holding potential of −120 mV for 250 ms. Two main voltage clamp protocols were used, as follows:

1. To assess the voltage dependence of activation of VGSCs and K^+^ channels, cells were held at −120 mV for 250 ms and then depolarised to test potentials in 5–10 mV steps between −120 mV and +30 mV for 50 ms.
2. To assess the voltage dependence of steady-state inactivation, cells were held at −120 mV for 250 ms followed by prepulses for 250 ms in 5–10 mV steps between −120 mV and +30 mV and a test pulse to −10 mV for 50 ms.

### Pharmacology

Tetrodotoxin citrate (TTX, HelloBio HB1035) was diluted in sterile-filtered water to a stock concentration of 1 mM TTX/8.44 mM citrate and stored at −30 °C. The working concentration was 30 μM TTX/253 μM citrate. Ouabain octahydrate (Sigma O3125) was diluted in DMSO to a stock concentration of 50 mM and stored at −30 °C. The working concentration was 300 nM. Cariporide (Santa Cruz Biotechnology SC337619) was diluted in DMSO to a stock concentration of 50 mM and stored at −30 °C. The working concentration was 20 μM. Sodium iodoacetate (Acros Organics 170970250) was diluted in water and made up immediately before each experiment. The working concentration was 2 μM. Oligomycin (Santa Cruz Biotechnology SC201551) was diluted to a stock concentration of 10 mM in DMSO and stored at −30 °C. The working concentration was 1 μM. 3-Bromopyruvate (Apexbio B7922) was diluted in DMSO and stored at −20 °C. The working concentration range was 250 µM–1 mM. Veratridine was diluted in DMSO to a stock concentration of 50 mM and stored at −20 °C. The working concentration was 100 µM.

### RNA sequencing

Mice were housed (up to 4/cage) and were chosen at random for cell implantation ensuring that both cell types were represented within each cage/block. RNA was extracted from 12 xenograft tumours (6 control MDA-MB-231 tumours and 6 Na_v_1.5 knockdown MDA-MB-231 tumours; blinded to sample type) using TRIzol (Invitrogen), according to the manufacturer’s instructions. A sample size of 6/group was used for the RNA-seq experiments, in accordance with standard practice/recommendations [74]. RNA quality assessment, library preparation, and 150 bp short-read, paired-end sequencing were conducted by Novogene Europe (Cambridge, UK) with 1 µg of total RNA used for sequencing library construction with NEBNext Ultra RNA Library Prep Kit for Illumina (NEB, USA). Raw FastQ files were mapped using Bbsplit from the Bbtools 39.01 suite against Mm10 and hg38 [75] and ambiguous reads were excluded. The disambiguated hg38 reads were aligned and the count matrix was created using Rsubread v2.12.3 [76]. Differential gene expression was calculated using DESeq2 v2_1.38.1 [77]. GO enrichment analysis was performed using clusterProfiler v4.6.2 [78]. GSEA was performed using the implementation in VULCAN v1.20.0 [79]. All code for the analysis is available from https://github.com/andrewholding/RNASeq-SCN5A.

### pH-selective microelectrodes

Unfilamented borosilicate capillary tubes were pulled to a resistance of ~5 MΩ (measured after silanization and when filled with PSS and in a recording bath). Silanization was performed at 200 °C for 15 min with *N*,*N*-dimethyltrimethylsilyamine. Microelectrodes were back filled with the following solution (in mM): NaCl 100, HEPES 20, NaOH 10, adjusted to pH 7.5. Microelectrodes were then front-filled with H^+^ ionophore I – cocktail A (Sigma) [80]. Recordings were made using a MultiClamp 900A amplifier (Molecular Devices) linked to a computer running MultiClamp 900A Commander software (Molecular Devices). The headstage amplifier was a high impedance 0.0001MU Axon HS-2 (Molecular Devices). Currents were digitised using an ITC018 A/D converter (HEKA Instruments), regular oscillatory noise was reduced with a HumBug noise eliminator (Quest Scientific) and the voltage signal was low-pass filtered at 10 Hz. Voltage was recorded using Axograph software (version 1.7.6). Electrodes were calibrated and offset from junction potentials empirically measured every 12 measurements to avoid drift with repeated electrode placement. A straight line was fitted to the offset-corrected voltage/pH calibration points, and the equation of this straight line was used to calculate the corresponding tissue slice pH. Tissue slices were maintained at 30 °C and 100% humidity in the recording chamber at the interface between air and perfused PSS. Measurements were made on the top surface of tumour tissue slices within 1 h of euthanasia. In total, 12 measurements were made from each region of the slice, alternating between regions, with calibrations and bath measurements taken before, half-way through and at the end of the series of measurements.

### Immunohistochemistry

Tissue cryopreservation, sectioning, and immunohistochemistry were performed as described previously [11]. The following primary antibodies were used: rabbit anti-active caspase 3 (1:200; R&D Systems AF835), and rabbit anti-Ki67 (1:5000; Abcam AB15580). The secondary antibody was Alexa-568 conjugated goat anti-rabbit (1:500; Invitrogen A11036). Sections were mounted in Prolong Gold + DAPI (Thermo Fisher). Stained sections were imaged on a Zeiss AxioScan.Z1 slide scanner at 20×. Images were viewed using Zen 3.4 (blue edition) software (Zeiss) and the maximal intensity in the red and blue (DAPI) channels was changed to maximise the visibility of positively stained cells. The minimum intensity was not changed. Images were then converted from .czi format to 8-bit .tif format. In ImageJ, a whole section was viewed at a time and the shape was matched to the drawing of the tissue slice during pH-selective microelectrode recording. Regions of interest (ROIs; 1000 × 1000 pixels) were chosen in both ‘core’ and ‘peripheral’ regions identified during recording. Six ROIs were selected from each region. Image analysis was performed using an ImageJ macro. Briefly, a nuclear count was performed by a particle count in the DAPI channel. A minimum intensity threshold was applied to the Alexa-568 channel, and this value was kept consistent within all ROIs from each tissue section. Activated caspase 3 staining was assessed by a particle count. Nuclear Ki67 staining was quantified by a particle count where the DAPI signal was colocalised with the Alexa-568 signal. Particle counts were expressed as a percentage of the DAPI particle count in the same ROI to give a percentage of positively stained cells in each ROI for each antibody and averaged to give a single value for each section (one section per tumour).

### Measurement of intracellular pH

Cells were grown on glass coverslips for 48 h, then were incubated for 10 min at 21 °C in 1 μM 2′,7′-bis(2-carboxyethyl)-5[6]-carboxyfluorescein acetoxymethyl ester (BCECF-AM, Biotium) in PSS, washed twice then left in PSS at pH 7.2 for 30 min before incubation for 10 min in PSS at pH 6.0 or 7.2. Coverslips were mounted in a Warner RC-20H recording chamber used in an open configuration at room temperature with PSS perfusion at 1 ml/min. Two-point calibration was performed at the end of every experiment using K^+^-based PSS (where Na^+^ was replaced by K^+^) at pH 7.0 with 13 μM nigericin (Sigma) for 7 min, followed by K^+^-based PSS at pH 8.0 for a further 7 min. For each individual cell, a standard curve was derived using these buffers of known pH value, and the resting pH calibrated. Exposures of 0.15 s duration were taken every 15 s with a Nikon Eclipse TE200 epi-fluorescence microscope using SimplePCI 6.0 software to control the imaging system. Images were captured with a RoleraXR Fast1394 CCD camera (Q-imaging) with a 10X Plan Fluor objective. Images were saved as 16-bit .tif files and analysed in ImageJ 1.53c. Circular ROIs were placed over cells. The mean intensity at each wavelength was calculated for each ROI. Background fluorescence was calculated for each excitation wavelength by selecting an ROI containing no cells. Background fluorescence was subtracted from the mean intensity of each ROI before fluorescence ratio calculation. Each experimental repeat was the mean measurement from ~40 cells/coverslip.

### Measurement of intracellular [Na^+^]

Cells were seeded at 2 × 10^4^ (MCF7) or 2.5 × 10^4^ (MDA-MB-231) cells/well in a 96-well, black-walled, µclear polymer-bottomed plate (Greiner 655097). Medium was exchanged and drug incubations started after 36 h. Before dye loading, wells were washed with PBS, and 60 μl DMEM containing SBFI-AM (10 μM) and Pluronic F-127 (0.1%) ± drug treatment was added to each well. Cells were incubated in SBFI-AM at 37 °C for 2 h. Wells were then washed twice in PSS ± drug and left in PSS ± drug for imaging on a BMG Clariostar plate reader with excitation at 340 and 380 nm and emission collected at 510 nm. Background fluorescence was subtracted from each wavelength before fluorescence ratio calculation. Each experimental repeat was the mean fluorescence ratio of five wells from a single plate.

### ATP quantification

CellTiter-Glo cell viability assay was used to measure cellular ATP levels following the manufacturer’s protocol (Promega, UK). Cells (1 × 10^4^/well) were cultured in 96-well plates for 24 h prior to incubation in glucose-free PSS (where glucose was replaced by choline to maintain osmolarity) for 90 min ± drug treatment (100 µM veratridine or DMSO vehicle). Following the 90-min incubation, 100 µl of CellTiter-Glo reagent was added to each well and plates were placed on an orbital shaker for 2 min to induce cell lysis, followed by incubation at room temperature for 10 min. Luminescence readings were then taken using a BMG Clariostar plate reader. Background luminescence was subtracted using wells containing reagents without cells. Each experimental repeat was the mean of five wells from a single plate.

### Viability assay

Cells were cultured in 6 well plates. Unless otherwise stated, culture medium from each well was removed into 14 ml Falcon tubes then adherent cells were detached using trypsin–EDTA and added to the same tube. Cells were centrifuged at 800 × *g* for 5 min and resuspended in medium. A 10 μl sample was mixed with an equal volume of trypan blue and the number of viable and dead cells was counted using an Invitrogen Countess automated cell counter.

### Metabolic profiling

OCR and ECAR measurements were conducted using a Seahorse XFe96 Extracellular Flux Analyzer (Agilent), based on methods described previously [34, 81]. The day prior to the measurements, MCF7 (2.0 × 10^4^/well) and MDA-MB-231 cells (3.0 × 10^4^/well) were seeded in a Seahorse XF96 cell culture microplate (101085-004, Seahorse) in 100 μl DMEM supplemented with 5% FBS and left at room temperature for 1 h. Cells were then transferred to a 5% CO_2_ incubator set at 37 °C and incubated overnight. The next day, cells were washed twice with 200 μl of assay medium (DMEM powder, D5030, Sigma; resuspended in 1 L UF water, adjusted to pH 7.4, sterile-filtered and supplemented on the day of the experiment with 17.5 mM glucose, 2 mM glutamine, and 0.5 mM sodium pyruvate) and a final volume of 180 μl was left per well. Then, the plate was incubated for equilibration in a 37 °C non-CO_2_ incubator for 1 h. In the meantime, the sensor cartridge was loaded with 20 μl of a 10× concentrated TTX solution to give a final assay concentration in the well of 30 μM. The experimental protocol consisted of consecutive cycles of 6 min that included 2 min of mixing followed by 4 min of measuring OCR and ECAR. After calibration of the cartridge and equilibration of the cell plate in the Seahorse Analyzer, basal measurements were acquired for six cycles, followed by the injection of the TTX or water vehicle control and measurement for ten more cycles. There were six replicate wells/plate and the experiment was repeated independently three times.

### Human breast cancer tissue microarray

A TMA consisting of formalin-fixed, paraffin-embedded cores from 1740 unselected primary operable invasive breast tumours held in Nottingham, UK was obtained from the BCNTB. The TMA series contains samples from patients age ≤71 years, treated in Nottingham University Hospitals NHS Trust according to standard clinical protocols, including different subtypes, histological grades, lymph node statuses, and treatment histories (including endocrine therapy and chemotherapy) [82]. Clinicopathological features of the series are summarised in Table 1. Immunohistochemistry was performed as described previously [83]. Samples were incubated with rabbit anti-human Na_v_1.5 antibody (1:100; Alomone ASC-013, which recognises residues 1978–2016 of human Na_V_1.5) and stained with EnVision+ Dual Link System/HRP (DAB+; Dako), following the manufacturer’s instructions and counterstained with Mayer’s haematoxylin. The specificity of staining was evaluated using antibody pre-adsorbed with immunising peptide. Slides were imaged on a Zeiss AxioScan.Z1 slide scanner with a 20× objective and staining was visualised using Zen 3.4 (blue edition) software (Zeiss). Staining was quantified using a modification to the Allred scoring system, as described previously [11, 84]. Briefly, the proportion of Na_v_1.5-positive cancer cells in each core was scored (none: 0; <1/100: 1; 1/100–1/10: 2; 1/10–1/3: 3; 1/3–2/3: 4; >2/3: 5), followed by a staining intensity score (none: 0; weak: 1; intermediate: 2; strong: 3). Next, the proportion and intensity scores were added to give an overall score of 0–8. TKL did the scoring and 10% of the series was independently verified by WF. In both cases, scoring was performed without prior knowledge of the associated clinical data. Concordance between investigators was assessed using Cohen’s weighted kappa (0.814; 95% CI 0.766–0.865; *P* < 0.001) and intraclass correlation coefficient (0.954; 95% CI 0.938–0.966; *P* < 0.001), indicating excellent agreement between scorers.

### Statistical analysis

Linear regression was used for the calibration of ratiometric indicators and pH electrodes over the recording ranges used. Statistical analysis was performed on raw (non-normalised) data using GraphPad Prism 9 for most analyses. IBM SPSS Statistics 27 was used to compute the intraclass correlation coefficient, Cohen’s weighted kappa and perform Cox multivariate proportional hazard analysis. No power calculation for sample sizes was performed as there is no relevant prior study on which this could be based. Pairwise statistical significance was determined with two-sided Student’s paired or unpaired, or one-sample *t* tests for normally distributed data and Mann–Whitney tests for non-parametric data. Multiple comparisons were made using ANOVA and Tukey’s or Dunnett’s multiple comparisons tests for normally distributed data and using Kruskal–Wallis or Friedman’s tests for non-parametric data. Outward current and Seahorse data were analysed using two-way ANOVA. Time-to-event data were analysed using Kaplan–Meier plots and log-rank (Mantel–Cox) tests computed with hazard ratio (HR) and 95% confidence intervals (CI). Correlations between Na_v_1.5 and other protein markers previously quantified in the same TMA [85] were evaluated using Spearman’s test. The correlation of *SCN5A* mRNA with ER activity in the TCGA breast cancer dataset (https://www.cancer.gov/tcga) was performed using VIPER [86]. Results were considered significant at *P* < 0.05 or Benjamini–Hochberg (BH) adjusted *P* < 0.05.

### Supplementary information

Supplementary Data

## Data Availability

The RNA-seq data are deposited in the GEO database, accession number GSE228621.

## Code availability

The code used to analyse the data is available from https://github.com/andrewholding/RNASeq-SCN5A.
