# Supplementary material for: A novel Nav1.5-dependent feedback mechanism driving glycolytic acidification in breast cancer metastasis
Source: Oncogene. 2024 Jul 25;43(34):2578–94. doi: 10.1038/s41388-024-03098-x (PMC11329375; doi:10.1038/s41388-024-03098-x)
Supplement: Supplementary file 1 — Supplementary Data [file 41388_2024_3098_MOESM1_ESM.pdf]

SUPPLEMENTARY DATA

A novel $Na_v1.5$-dependent feedback mechanism driving glycolytic acidification in breast cancer metastasis

Theresa K Leslie[1,2], Aurelien Tripp[3], Andrew D James[1,2], Scott P Fraser[4], Michaela Nelson[1,2], Nattanan Sajjaboontawee[1,2], Alina L Capatina[1,2], Michael Toss[5], Wakkas Fadhil[5], Samantha C Salvage[8], Mar Arias Garcia[3], Melina Beykou[3,10], Emad Rakha[5], Valerie Speirs[6], Chris Bakal[3], George Poulogiannis[3], Mustafa B A Djamgoz[4,7], Antony P Jackson[8], Hugh R Matthews[8], Christopher L-H Huang[8,9], Andrew N Holding[1,2], Sangeeta Chawla[1,2], and William J Brackenbury[1,2]

[1] Department of Biology, University of York, York, UK

[2] York Biomedical Research Institute, University of York, York, UK

[3] Division of Cancer Biology, Institute of Cancer Research, London, UK

[4] Department of Life Sciences, Imperial College London, London SW7 2AZ, UK

[5] Department of Pathology, School of Medicine, University of Nottingham, NG5 1PB, UK

[6] Institute of Medical Sciences, University of Aberdeen, Aberdeen, AB24 2ZD, UK

[7] Biotechnology Research Centre, Cyprus International University, Haspolat, TRNC, Mersin 10, Türkiye

[8] Department of Biochemistry, University of Cambridge, Cambridge, UK

[9] Physiological Laboratory, University of Cambridge, Cambridge, UK

[10] Department of Electrical and Electronic Engineering, Imperial College London, London, SW7 2AZ, UK

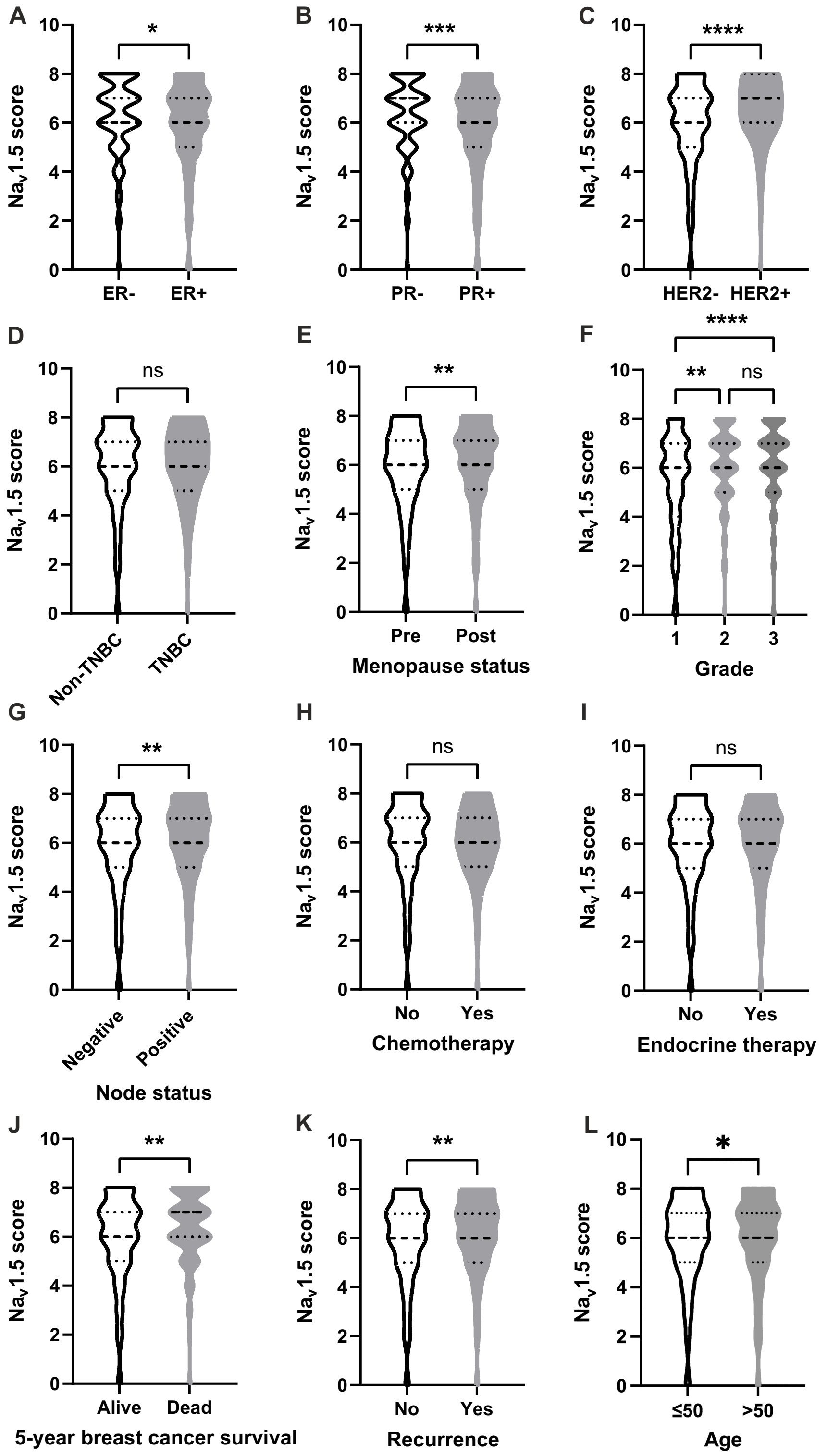

Supplementary Figure 1. Na$_V$1.5 staining intensity score compared across different histoclinical groups. (A) Estrogen receptor (ER)+ vs. ER-. (B) Progesterone receptor (PR)+ vs. PR-. (C) Human epidermal growth factor 2 (HER2)+ vs. HER2-. (D) Triple-negative breast cancer (TNBC) vs. non-TNBC. (E) Pre- vs. post-menopausal. (F). Tumour grade. (G) Lymph node status. (H) Treated or not treated with chemotherapy. (I) Treated or not treated with endocrine therapy. (J) Five-year survival status. (K) Recurrence status. (L) Age at diagnosis. Data are median ± quartiles. ****P < 0.0001, ***P < 0.001, **P < 0.01, *P < 0.05, ns, not significant. Mann-Whitney tests for all except (F): Kruskal-Wallis with Dunn's test.

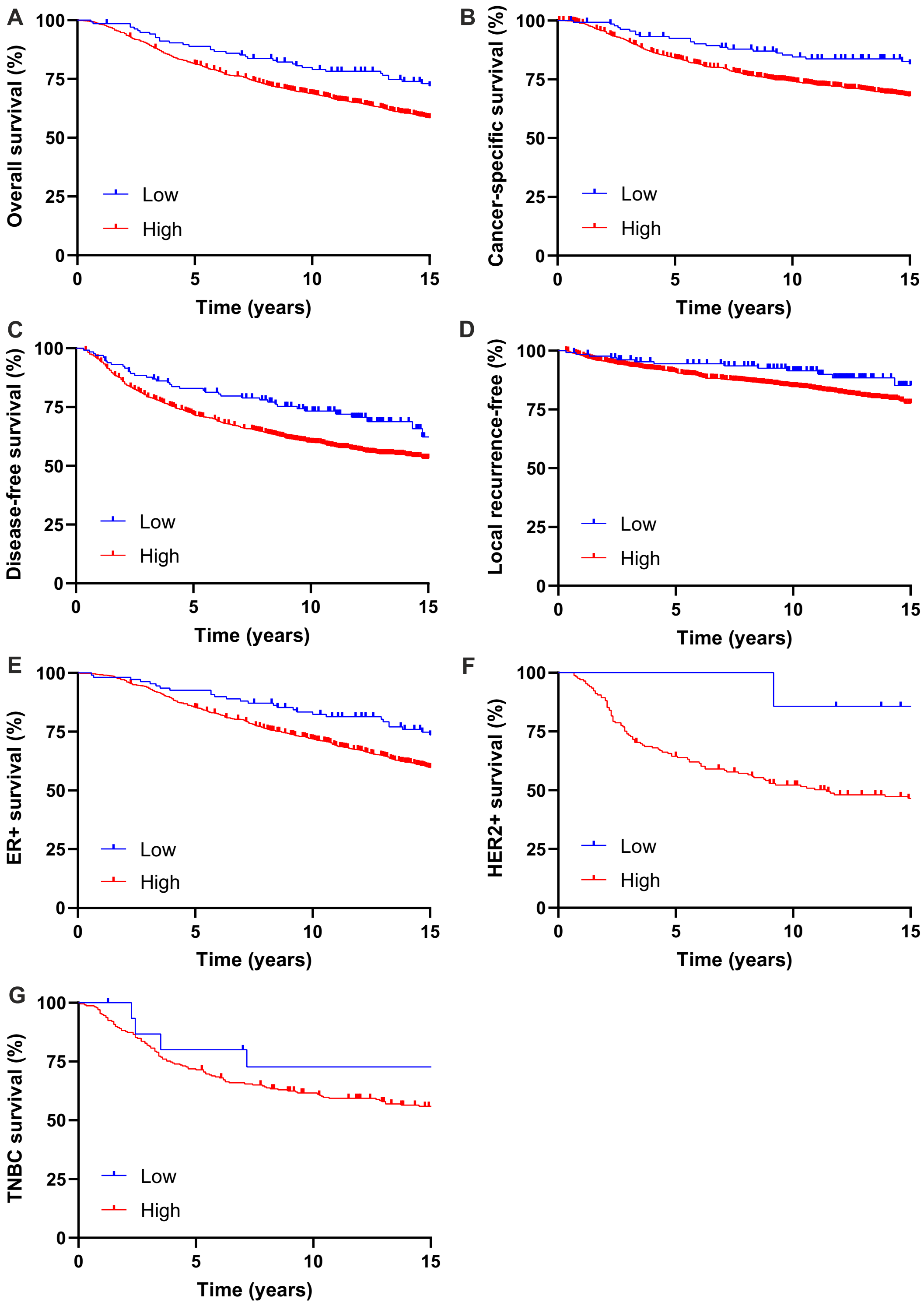

Supplementary Figure 2. Survival of breast cancer patients high or low $Na_v1.5$-expressing tumours. (A) Overall survival; HR = 1.44 (95% CI 1.12-1.84); $P < 0.05$ (log-rank test). (B) Cancer-specific survival; HR = 1.87 (95% CI 1.38-2.53); $P < 0.01$ (log-rank test). (C) Disease-free survival; HR 1.56 (95% CI 1.19-2.04); $P < 0.01$ (log-rank test). (D) Local recurrence-free survival; HR = 1.86 (95% CI 1.21-2.86); $P < 0.05$ (log-rank test). (E) Overall survival of patients with estrogen receptor (ER)+ tumours; HR = 1.51 (95% CI 1.14-1.99); $P < 0.05$ (log-rank test). (F) Overall survival of patients with human epidermal growth factor 2 (HER2)+ tumours; HR = 2.71 (95% CI 1.12-6.52); P = 0.14 (log-rank test). (G) Overall survival of patients with triple-negative breast cancer (TNBC); HR = 1.30 (95% CI 0.62-2.70); P = 0.53 (log-rank test).

Supplementary Table 1. Correlation between $Na_v1.5$ and other protein markers.

**Protein marker**	**Spearman's correlation coefficient**	**BH-adjusted P value**	**n**
ALDH1A1	-0.05	0.328	579
AMPK	-0.15	<0.001	1214
Carbonic anhydrase IX	-0.07	0.170	479
Cathepsin D	0.11	0.007	762
CD44	-0.13	0.003	701
CD56	-0.19	0.001	372
HIF-1	-0.05	0.651	130
IDH2	0.13	0.001	715
Kank1M	0.08	0.113	546
Lactate dehydrogenase 5	0.00	0.942	434
MMP11	-0.02	0.883	117
MMP13 (cytoplasmic)	-0.02	0.561	977
MMP13 (nuclear)	-0.20	<0.001	977
MMP14 (cytoplasmic)	-0.07	0.057	868
MMP14 (membrane)	-0.09	0.016	867
MMP14 (nuclear)	0.01	0.817	867
MMP15 (cytoplasmic)	-0.05	0.259	842
MMP-15 (membrane)	0.19	<0.001	842
MMP15 (nuclear)	0.19	<0.001	842
MMP2 (cytoplasmic)	-0.20	<0.001	847
MMP2 (nuclear)	-0.10	0.007	847
MMP7	-0.15	<0.001	866
MMP9	0.11	0.015	599
MX1	0.05	0.227	741
NHERF1	0.07	0.278	321
SNAT2	-0.12	0.011	593

Supplementary Table 2. Receptor status of patient tumour tissue slices

Sample	Receptor status
Patient 1	ER-<br>HER2+
Patient 2	ER+<br>HER2-
Patient 3	ER+<br>HER2-

Supplementary Table 3. Characteristics of primary cell samples

Sample	Type	Receptor status
2357N	Normal	n/a
4800N	Normal	n/a
2252N	Normal	n/a
2585N	Normal	n/a
2380T	Tumour	ER+ HER2+
1691T	Tumour	ER+ HER2+
2360T	Tumour	TNBC
2285T	Tumour	ER+ HER2+
1704T	Tumour	HER2+
1314T	Tumour	ER+ HER2+
2615T	Tumour	ER+ HER2+
1958T	Tumour	TNBC
2724T	Tumour	ER+ HER2+
2599T	Tumour	TNBC
1997T	Tumour	TNBC
2424T	Tumour	TNBC
2753T	Tumour	TNBC

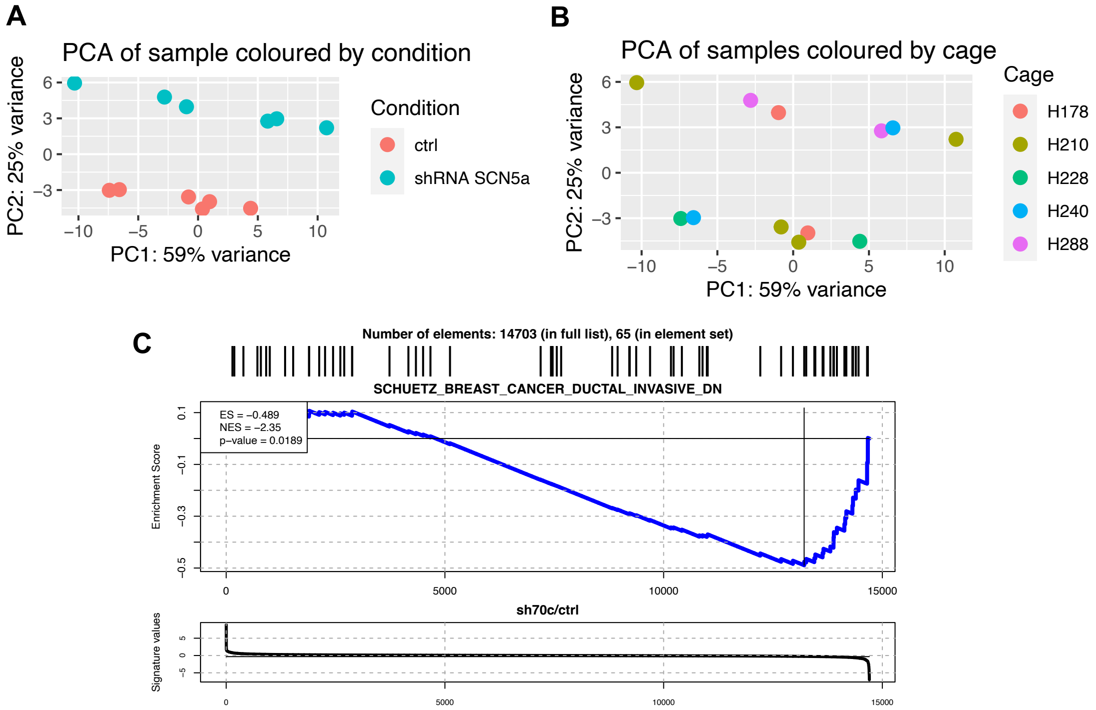

Supplementary Figure 3. Transcriptional response to *SCN5A* downregulation in xenograft tumours. (A) Principal component analysis (PCA) biplot of tumour sample coloured by cell type (shControl vs shRNA *SCN5A*). (B) PCA biplot of tumour sample coloured by animal cage. (C) Gene set enrichment analysis (GSEA) of invasion-downregulated genes defined in the MSigDB gene set SCHUETZ_BREAST_CANCER_DUCTAL_INVASIVE_DN analysed in $Na_v1.5$ knockdown vs. control tumours.

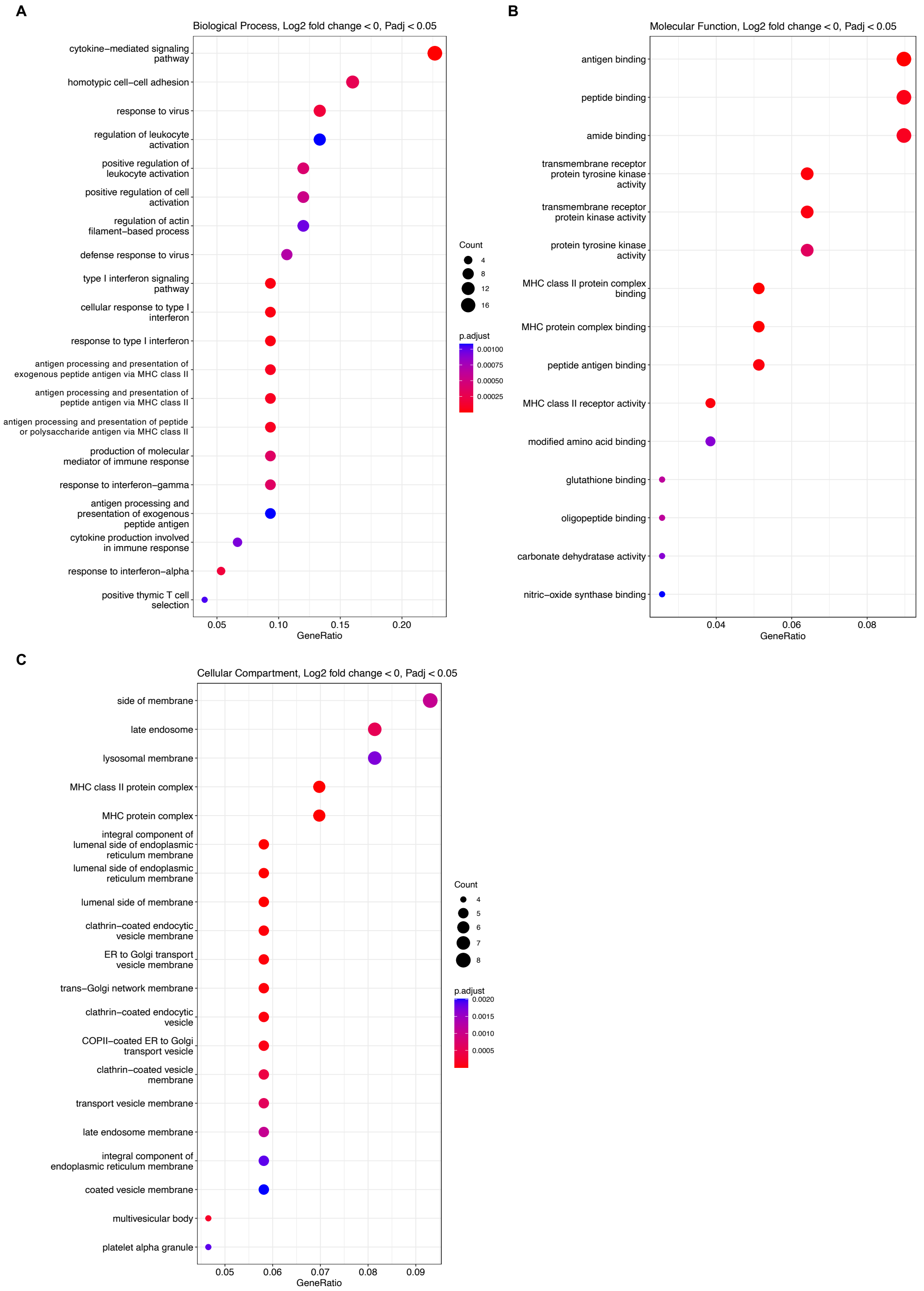

Supplementary Figure 4. Enrichment of GO terms in differentially expressed genes in *SCN5A* knock-down tumours for (A) Biological process, (B) Molecular function, and (C) Cellular compartment.

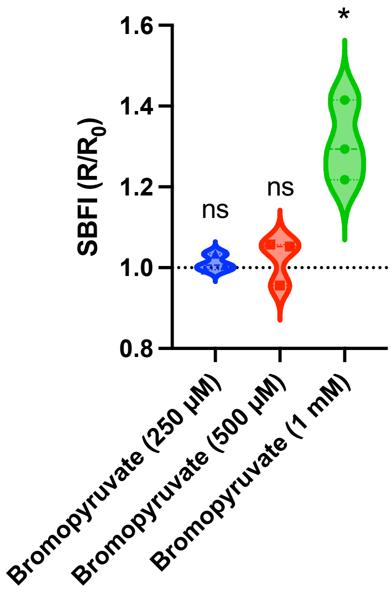

Supplementary Figure 5. Effect of 3-bromopyruvate on $[Na^+]_i$ of MDA-MB-231 cells. (A) SBFI-AM fluorescence ratios of MDA-MB-231 cells after 2 h treatment with 3-bromopyruvate at the indicated concentrations. Data are normalised to the ratio in vehicle-treated cells (0.6% DMSO). *P < 0.05; ns, not significant (n = 3 experimental repeats; one sample *t* tests).

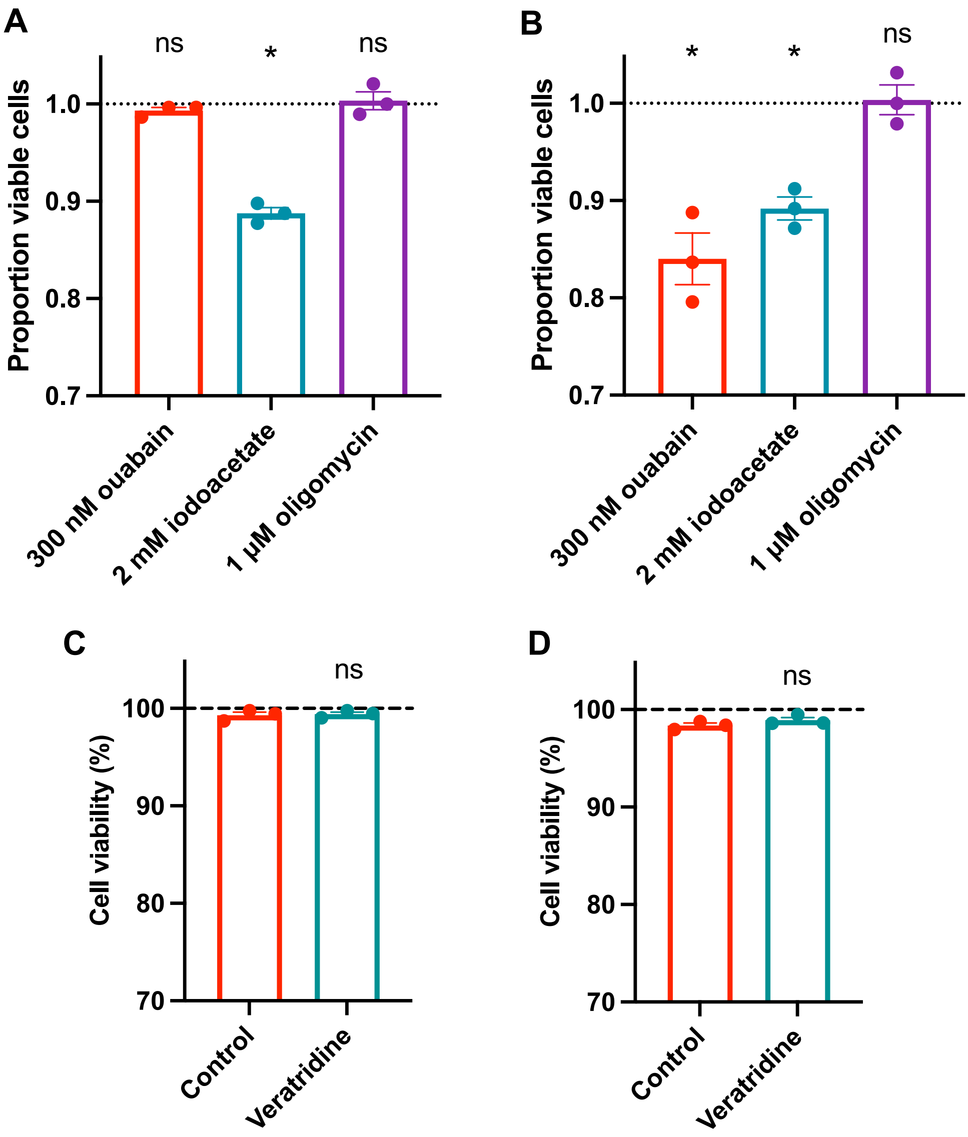

Supplementary Figure 6. Effect of glycolytic and mitochondrial respiration on viability of MDA-MB-231 and MCF7 cells. (A) Viability of MDA-MB-231 cells after 6 h treatment with ouabain (300 nM), or 2 h treatment with sodium iodoacetate (2 mM) or oligomycin (1 μM). Data are normalised to the ratio in vehicle-treated cells (n = 3 experimental repeats; one sample *t* tests). (B) Viability of MCF7 cells after 6 h treatment with ouabain (300 nM) or 2 h treatment with sodium iodoacetate (2 mM) or oligomycin (1 μM). Data are normalised to the ratio in vehicle-treated cells (n = 3 experimental repeats; one sample *t* tests). (C) Viability of MDA-MB-231 cells after 90 min treatment in Na^+-free physiological saline solution (PSS) with veratridine (100 μM) or DMSO vehicle (n = 3 experimental repeats; *t* tests). (D) Viability of MCF7 cells after 90 min treatment in Na^+-free PSS with veratridine (100 μM) or DMSO vehicle (n = 3 experimental repeats; *t* tests). For (C) and (D), viability was assessed using the trypan blue assay method described previously (1). Data are mean ± SEM. *P < 0.05, ns, not significant.

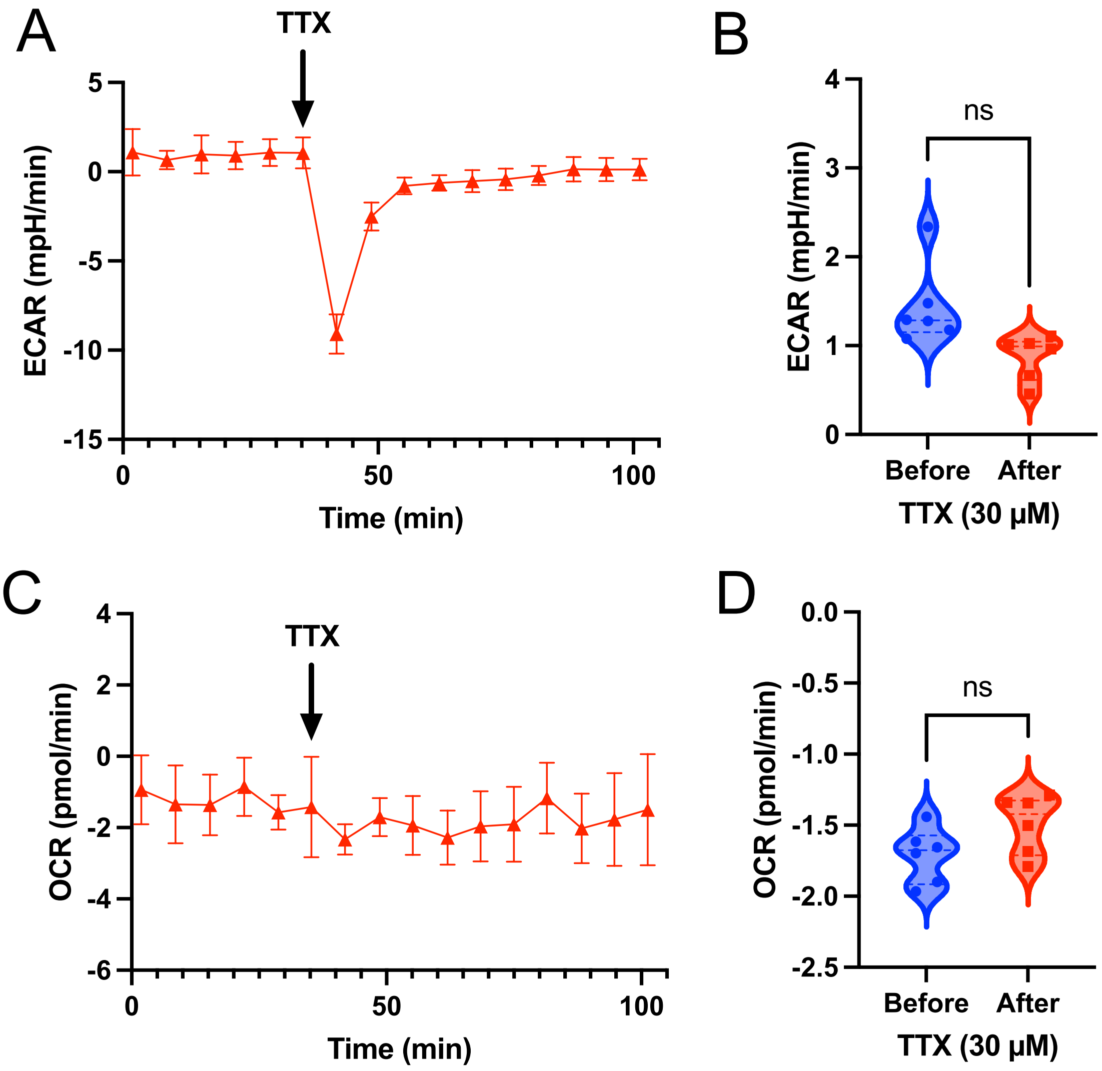

Supplementary Figure 7. Effect of TTX citrate buffering on measurements of extracellular acidification rate (ECAR) and oxygen consumption rate (OCR). (A) Representative measurements of ECAR in wells containing medium only (no cells). TTX was added to the treated cells at the indicated timepoint, to give a final concentration of 30 µM (n = 3 wells). (B) ECAR compared before (first 6 timepoints) vs. after TTX addition (last 6 timepoints). (C) Representative measurements of OCR in wells containing medium only. TTX was added to the treated cells at the indicated timepoint, to give a final concentration of 30 µM (n = 3 wells). (D) OCR compared before (first 6 timepoints) and after TTX addition (last 6 timepoints). Data are mean ± SEM. ns, not significant (2-way ANOVA).

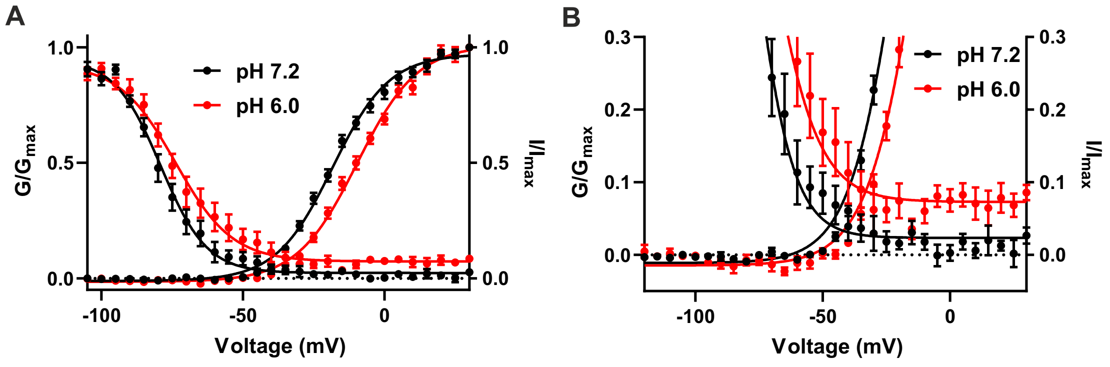

Supplementary Figure 8. Effect of pH$_e$ 6.0 on VGSC gating in MDA-MB-231 cells. (A) Overlay of activation and inactivation curves at pH 7.2 and 6.0 (n = 8 cells with the largest currents). (B) Expanded data from (A) showing the window current. Data are mean ± SEM.

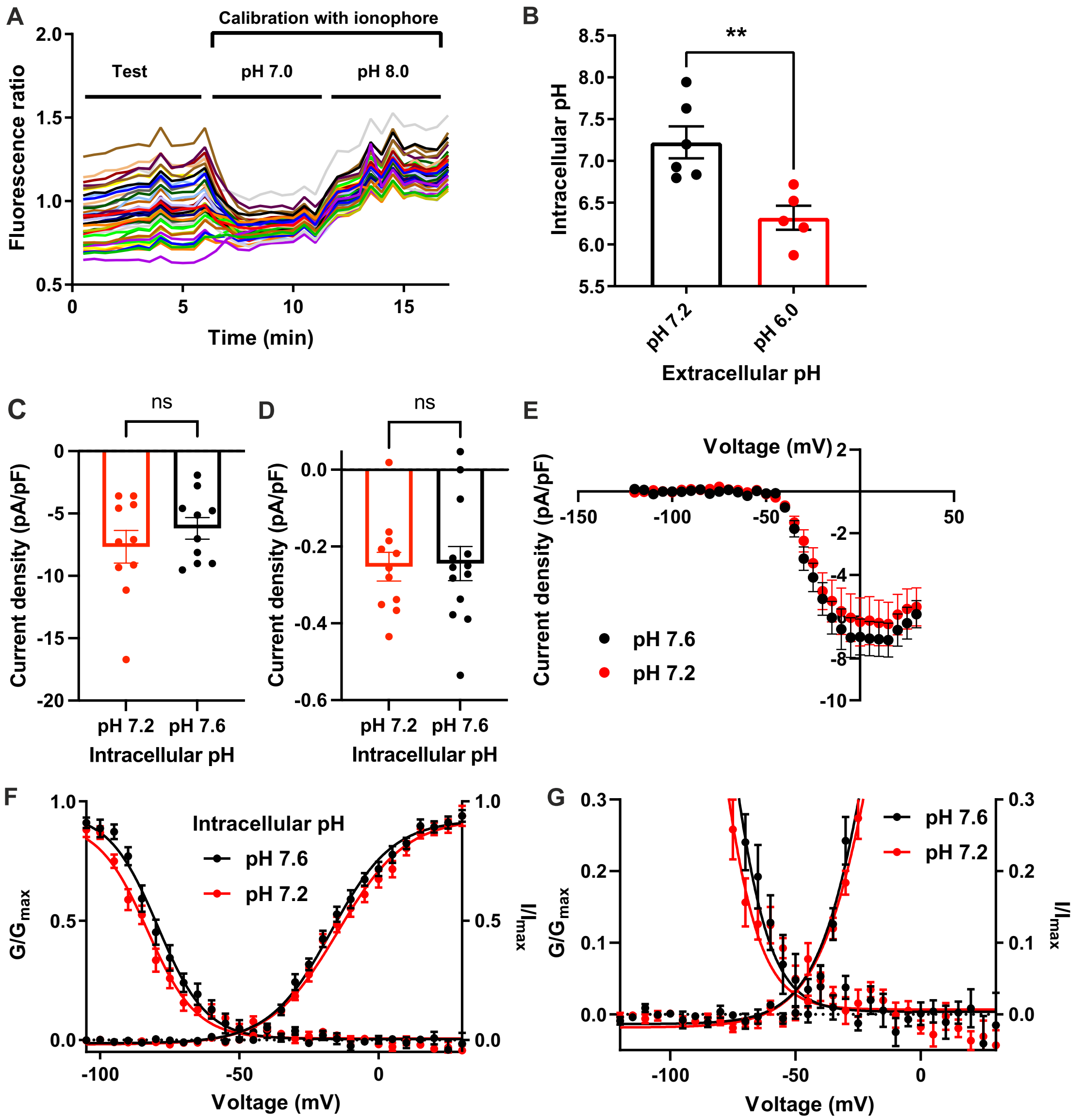

Supplementary Figure 9. Effect of intracellular pH on VGSC currents and gating. (A) Example two-point calibration of BCECF-AM fluorescence ratio in MDA-MB-231 cells on a coverslip (each coloured line is one cell). (B) Effect of 10-minute incubation with physiological saline solution at pH 7.2 or 6.0 on pH_i in MDA-MB-231 cells (n ≥ 5 experiments of 40 cells each; unpaired *t* test). (C) Peak Na^+ current density at pH 7.2 and pH 7.6 in patch pipette (n = 10 cells, unpaired *t* test). (D) Mean persistent Na^+ current density measured 20-25 ms following depolarisation (n = 11-13 cells; unpaired *t* test). (E) Average current density/voltage relationship at pH_i 7.2 or 7.6 (n = 10-12 cells). (F) Activation and steady-state inactivation curves at pH_i 7.2 and 7.6 (n = 10-12 cells). (G) Expanded graph from (F) showing the window current, which is unchanged between pH_i of 7.2 and 7.6. Data are mean ± SEM. **P < 0.01, ns, not significant.

Calculation to predict Na_v1.5-dependent extracellular acidification rate

Assumptions:

Persistent Na^+ current into breast cancer cells through VGSCs is counteracted by activity of NKA to maintain a stable $[Na^+]_i$. NKA pumps 3 Na^+ ions out of the cell per cycle in which it hydrolyses one molecule of ATP (2). Glycolytic production of lactic acid produces 2 molecules of ATP and 2 molecules of lactate per glucose. The production of H^+ by this reaction coupled to the ATPase hydrolysis of ATP to ADP generates 2 H^+ per glucose (3). There is therefore net production of one H^+ per ATP molecule generated by glycolytic fermentation to produce lactate.

Therefore:

(H^+ evolution rate) = (Na^+ influx rate)/3 *[1]*

It is also assumed that all H^+ produced is removed via NHE1 to maintain a stable intracellular pH:

(H^+ evolution rate) = (NHE1 rate) *[2]*

Since this extra NHE1 activity leads to further Na^+ entry (1:1 exchange of Na^+ for H^+):

(Na^+ influx rate) = {(Na_v1.5 persistent current) / F} + (NHE1 rate) *[3]*

where F = Faraday constant

So at intracellular steady state of $[H^+]$ and $[Na^+]_i$, substituting in *[3]* from *[1]* and *[2]*:

3 x (H^+ evolution rate) = {(Na_v1.5 persistent current) / F} + (H^+ evolution rate)

which is equivalent to:

(H^+ evolution rate) = {(Na_v1.5 persistent current)/(2 x F)}

Calculating the Na_v1.5 persistent current

Extracellular acidification through NHE1 due to Na_v1.5 activity happens in caveolae (4). The shape of these caveolae could provide a localised extracellular microenvironment with a particularly low pH. We showed earlier that in MDA-MB-231

cells at pH_e 6.0, the maximal transient Na^+ current density was -9.19 ± 1.28 pA/pF. The availability of channels at the resting membrane potential of -18.9 mV was 10.3 ± 2.2% of the maximal transient current (Figure 6J) so the persistent current density was -0.95 ± 0.13 pA/pF.

Na_v1.5 persistent current = -0.95 ± 0.13 pA/pF at pH 6.0

Whole cell capacitance = 26.0 ± 2.2 pF

Cell density for MDA-MB-231 cells in Seahorse analyzer: 3×10^4 / well

F = 96500 C/mol

Therefore:

Molar (H^+ evolution rate) per well = (Na_v1.5 persistent current density) x (Cell Capacitance) x (Cell density) /(2 x F)

Molar (H^+ evolution rate) per well = $(0.95 \times 10^{-12} \times 26 \times 3 \times 10^{4}) / (2 \times 96500)$

$$= 3.83 \times 10^{-12} \text{ mol/s}$$

Predicted pH change per second = (Molar H^+ evolution rate) / ((Well Volume) x (Buffer Capacity))

Well volume = 200×10^{-6} l

Buffer Capacity = 0.897×10^{-3} mol/l/pH (Supplementary Table 4)

Predicted pH change per second = $3.83 * 10^{-12} / (200 \times 10^{-6} \times 0.897 \times 10^{-3})$

$$= 2.13 \times 10^{-5} \text{ pH/s}$$

Predicted pH change per minute = $2.13 * 10^{-5} \times 60 = 1.28 \times 10^{-3}$ pH/min

$$= 1.3 \text{ mpH/min}$$

Supplementary Table 4. Calculation of buffering capacity of medium used in Seahorse analyzer experiments.

Buffer	Concentration	pKa	Ka	pH	Proton conc	Buffer factor
	M		M		M	moles/litre/pH
**Citrate**	2.53E-04	3.13	7.447E-04	7.4	3.981E-08	3.114E-08
	2.53E-04	4.76	1.734E-05	7.4	3.981E-08	1.332E-06
	2.53E-04	6.40	4.018E-07	7.4	3.981E-08	4.779E-05
**Glutamine**	2.00E-03	2.17	6.761E-03	7.4	3.981E-08	2.712E-08
	2.00E-03	9.13	7.413E-10	7.4	3.981E-08	8.266E-05
**Glycine**	4.00E-04	2.34	4.571E-03	7.4	3.981E-08	8.023E-09
	4.00E-04	9.60	2.512E-10	7.4	3.981E-08	5.740E-06
**Arginine**	4.00E-04	2.17	6.761E-03	7.4	3.981E-08	5.424E-09
	4.00E-04	9.04	9.120E-10	7.4	3.981E-08	2.017E-05
	4.00E-04	12.48	3.311E-13	7.4	3.981E-08	7.662E-09
**Cysteine**	2.00E-04	1.96	1.096E-02	7.4	3.981E-08	1.672E-09
	2.00E-04	10.28	5.248E-11	7.4	3.981E-08	6.056E-07
	2.00E-04	8.18	6.607E-09	7.4	3.981E-08	5.623E-05
**Histidine**	2.00E-04	1.82	1.514E-02	7.4	3.981E-08	1.211E-09
	2.00E-04	9.17	6.761E-10	7.4	3.981E-08	7.563E-06
	2.00E-04	6.00	1.000E-06	7.4	3.981E-08	1.696E-05
**Isoleucine**	8.00E-04	2.36	4.365E-03	7.4	3.981E-08	1.680E-08
	8.00E-04	9.60	2.512E-10	7.4	3.981E-08	1.148E-05
**Leucine**	8.00E-04	2.36	4.365E-03	7.4	3.981E-08	1.680E-08
	8.00E-04	9.60	2.512E-10	7.4	3.981E-08	1.148E-05
**Lysine**	8.00E-04	2.18	6.607E-03	7.4	3.981E-08	1.110E-08
	8.00E-04	8.95	1.122E-09	7.4	3.981E-08	4.912E-05
	8.00E-04	10.53	2.951E-11	7.4	3.981E-08	1.364E-06
**Methionine**	2.00E-04	2.28	5.248E-03	7.4	3.981E-08	3.494E-09
	2.00E-04	9.21	6.166E-10	7.4	3.981E-08	6.918E-06
**Phenylalanine**	4.00E-04	1.83	1.479E-02	7.4	3.981E-08	2.479E-09
	4.00E-04	9.13	7.413E-10	7.4	3.981E-08	1.653E-05
**Serine**	4.00E-04	2.21	6.166E-03	7.4	3.981E-08	5.948E-09
	4.00E-04	9.15	7.079E-10	7.4	3.981E-08	1.581E-05
**Threonine**	8.00E-04	2.09	8.128E-03	7.4	3.981E-08	9.024E-09
	8.00E-04	9.10	7.943E-10	7.4	3.981E-08	3.534E-05
**Tryptophan**	8.00E-05	2.83	1.479E-03	7.4	3.981E-08	4.959E-09
	8.00E-05	9.39	4.074E-10	7.4	3.981E-08	1.847E-06
**Valine**	8.00E-04	2.32	4.786E-03	7.4	3.981E-08	1.532E-08
	8.00E-04	9.62	2.399E-10	7.4	3.981E-08	1.097E-05
**Pyruvate**	5.00E-04	2.45	3.548E-03	7.4	3.981E-08	1.292E-08
**TTX**	3.00E-05	8.76	1.738E-09	7.4	3.981E-08	2.769E-06
					**OrganicSum**	**4.03E-04**
**NaH_2PO_4**	9.00E-04	2.16	6.918E-03	7.4	3.981E-08	1.193E-08
	9.00E-04	7.21	6.166E-08	7.4	3.981E-08	4.942E-04
	9.00E-04	12.32	4.786E-13	7.4	3.981E-08	2.492E-08
					**InorganicSum**	**4.94E-04**
**WaterTerm**				7.4	3.981E-08	2.910E-07
					**WaterTerm**	**2.91E-07**
					**TotalBufferCapacity**	**8.97E-04**

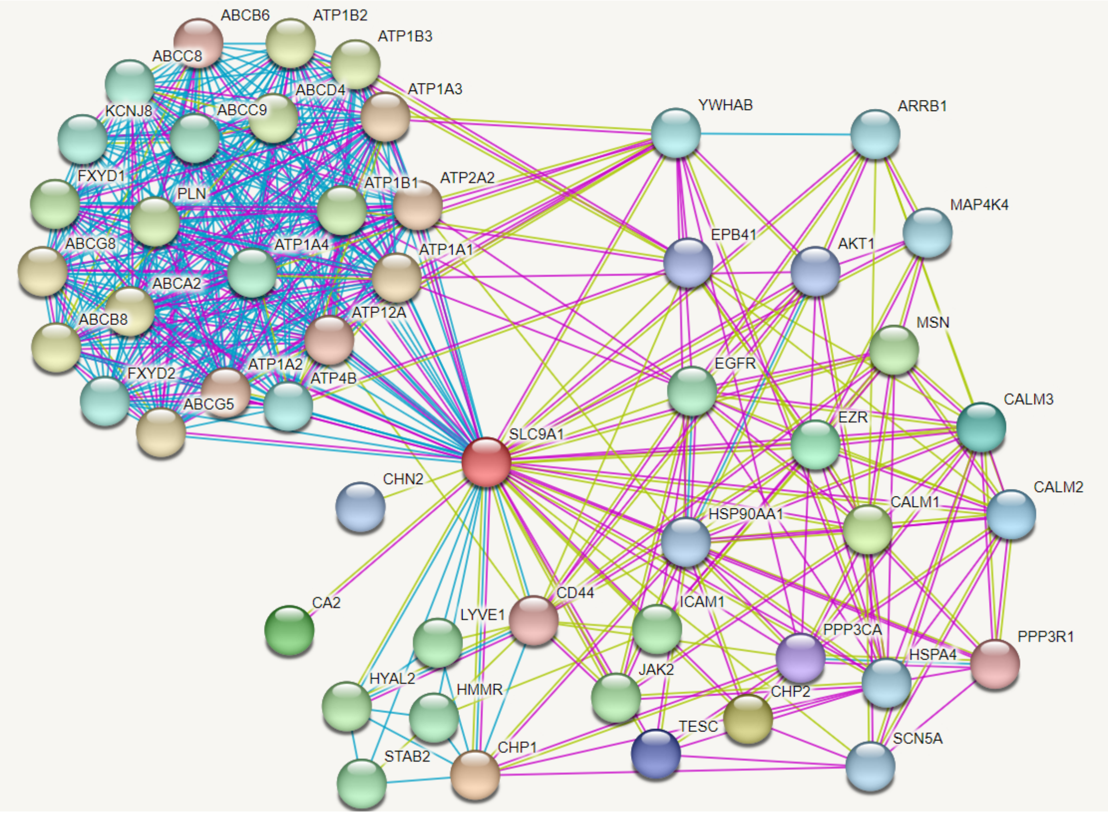

Supplementary Figure 10. *In silico* analysis of protein-protein interactions of NHE1 *(SLC9A1)*, created using the STRING database v11.5 © STRING consortium 2022. Only the physical subnetwork is shown (proteins must be part of a physical complex). Evidence for interactions is colour-coded: cyan = from curated databases, magenta = experimentally determined, pale green = text mining. Genes with names *ATP1A** or *ATP1B** are NKA subunits.

References

1. Yang M, Kozminski DJ, Wold LA, Modak R, Calhoun JD, Isom LL, et al. Therapeutic potential for phenytoin: targeting Na(v)1.5 sodium channels to reduce migration and invasion in metastatic breast cancer. Breast Cancer Res Treat. 2012;134(2):603-15.
2. Post RL, Jolly PC. The linkage of sodium, potassium, and ammonium active transport across the human erythrocyte membrane. Biochim Biophys Acta. 1957;25(1):118-28.
3. Hochachka PW, Mommsen TP. Protons and anaerobiosis. Science. 1983;219(4591):1391-7.
4. Brisson L, Driffort V, Benoist L, Poet M, Counillon L, Antelmi E, et al. NaV1.5 Na(+) channels allosterically regulate the NHE-1 exchanger and promote the activity of breast cancer cell invadopodia. J Cell Sci. 2013;126(Pt 21):4835-42.
